# ROR1 sustains caveolae and survival signalling as a scaffold of cavin-1 and caveolin-1

**DOI:** 10.1038/ncomms10060

**Published:** 2016-01-04

**Authors:** Tomoya Yamaguchi, Can Lu, Lisa Ida, Kiyoshi Yanagisawa, Jiro Usukura, Jinglei Cheng, Naoe Hotta, Yukako Shimada, Hisanori Isomura, Motoshi Suzuki, Toyoshi Fujimoto, Takashi Takahashi

**Affiliations:** 1 Division of Molecular Carcinogenesis, Center for Neurological Diseases and Cancer, Nagoya University Graduate School of Medicine, Nagoya 466-8550, Japan; 2 Division of Integrated Project, EcoTopia Science Institute, Nagoya University, Nagoya 464-8603, Japan; 3 Department of Anatomy and Molecular Cell Biology, Nagoya University Graduate School of Medicine, Nagoya 466-8550, Japan

## Abstract

The receptor tyrosine kinase-like orphan receptor 1 (ROR1) sustains prosurvival signalling directly downstream of the lineage-survival oncogene NKX2-1/TTF-1 in lung adenocarcinoma. Here we report an unanticipated function of this receptor tyrosine kinase (RTK) as a scaffold of cavin-1 and caveolin-1 (CAV1), two essential structural components of caveolae. This kinase-independent function of ROR1 facilitates the interactions of cavin-1 and CAV1 at the plasma membrane, thereby preventing the lysosomal degradation of CAV1. Caveolae structures and prosurvival signalling towards AKT through multiple RTKs are consequently sustained. These findings provide mechanistic insight into how ROR1 inhibition can overcome EGFR–tyrosine kinase inhibitor (TKI) resistance due to bypass signalling via diverse RTKs such as MET and IGF-IR, which is currently a major clinical obstacle. Considering its onco-embryonic expression, inhibition of the scaffold function of ROR1 in patients with lung adenocarcinoma is an attractive approach for improved treatment of this devastating cancer.

## Abstract (web summary)

Resistance to receptor tyrosine kinase inhibitors is a major obstacle in treatment of lung adenocarcinoma. Yamaguchi *et al.* identify the orphan receptor ROR1 as a potential target to overcome this resistance, by virtue of its role in promoting cell survival through stabilisation of caveolae.

## Graphical abstract

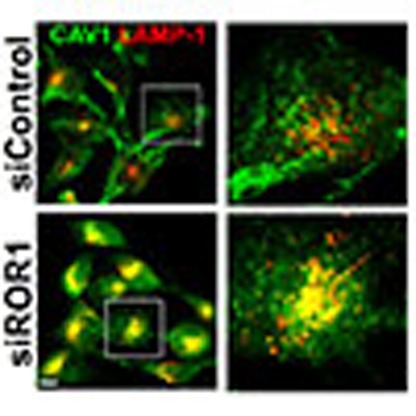

Caveolae are 50–100 nm invaginations of the plasma membrane that play various physiological roles^1^,^2^,^3^,^4^. Caveolin-1 (CAV1) is an essential structural component of caveolae, and cavin-1 (also known as PTRF), a soluble cytosolic protein, associates with CAV1 and prevents its lysosomal degradation^5^,^6^. This association enables CAV1 and cavin-1 to be stably confined to the plasma membrane, a process that is thought to be an indispensable prerequisite for caveolae formation. Caveolae have been suggested to function as a platform for insulin-induced signalling in adipose tissue^4^. However, the specific biochemical and physiological roles of caveolae remain to be fully elucidated for all relevant tissues^1^,^2^. The CAV1 mode of involvement appears to vary considerably among human cancers; however, CAV1 is generally thought to play a promoting role in the development of non-small cell lung cancers (NSCLCs)^7^,^8^,^9^.

Lung cancers have long been the leading cause of cancer death in economically advanced countries, with lung adenocarcinoma being the most frequent and steadily increasing lung cancer among NSCLCs. Receptor tyrosine kinases (RTKs) have been shown to be crucially involved in the molecular pathogenesis of NSCLCs, and epidermal growth factor receptor (EGFR)–tyrosine kinase inhibitors (TKIs) are widely used as an effective therapeutic option for patients with lung adenocarcinomas carrying mutant EGFR. However, the near-certain occurrence of treatment resistance remains a major obstacle^10^,^11^. Multiple mechanisms for EGFR–TKI resistance have been identified, including the secondary T790M EGFR mutation, as well as bypass signalling through other RTKs such as MET and insulin-like growth factor-I receptor (IGF-IR)^12^,^13^. Notably, such resistance-conferring events may arise within the same tumour undergoing EGFR–TKI treatment^14^, making it difficult to predict appropriate targets for the suppression and elimination of resistant clones.

We previously identified receptor tyrosine kinase-like orphan receptor 1 (ROR1) as a target for transcriptional activation via the lineage-survival oncogene NKX2-1/TTF-1 with frequent gene amplification and overexpression in lung adenocarcinoma^15^,^16^. ROR1 sustained PI3K-AKT signalling in part through ROR1 kinase-dependent c-Src activation, as well as the kinase activity-independent sustainment of EGFR–ERBB3 association through its extracellular domain and subsequent ERBB3 phosphorylation and PI3K activation. Interestingly, ROR1 knockdown effectively overcame the EGFR–TKI resistance conferred by hepatocyte growth factor (HGF)-mediated bypass signalling through MET, suggesting that ROR1 sustains signalling of not only EGFR but also other RTKs. However, the underlying mechanism was elusive.

In this study, we aimed to elucidate how ROR1 sustains signalling for multiple RTKs in NSCLCs. We consequently discovered an unanticipated function of this RTK. We found that ROR1 functions as a scaffold protein of cavin-1 and CAV1, two essential structural components of caveolae, a function that in turn sustains caveolae formation and prosurvival signalling through multiple RTKs in NSCLC cells.

## Results

### Reduced phosphorylation of multiple RTKs by siROR1 or siCAV1

**Figure 1:**
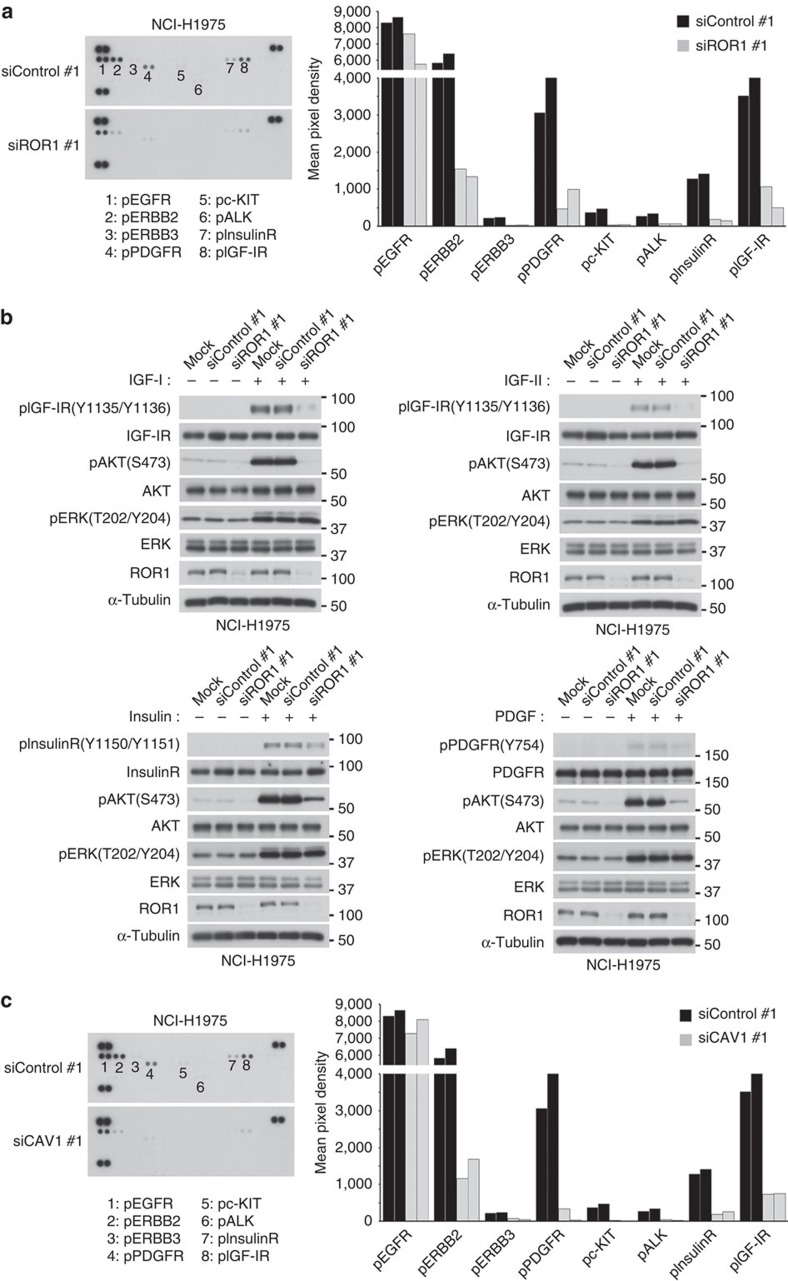
ROR1 and CAV1 knockdown results in decreased phosphorylation of multiple RTKs. (**a**) Phospho-RTK array results showing the inhibitory effects of siROR1 treatment on the phosphorylation state of multiple RTKs in NCI-H1975 cells (left panel). Averages of the mean pixel densities in two independent experiments are given for each of the representative RTKs (right panel). See Supplementary Fig. 1a for data in PC-9 cells. (**b**) The impairment of growth factor-induced phosphorylation in multiple RTKs in NCI-H1975 cells knocked down for ROR1. See Supplementary Fig. 1b for data in PC-9 cells. (**c**) Phospho-RTK array results showing the inhibitory effects of siCAV1 treatment on the phosphorylation state of multiple RTKs in NCI-H1975 cells (left panel), and averages of the mean pixel densities of the representative RTKs in two independent experiments (right panel). The siControl blot of **a** is re-displayed for ease of comparison. See Supplementary Fig. 1c for data in the PC-9 NSCLC cell line. Uncropped images of blots are shown in Supplementary Fig. 11.

We first analysed the effects of siROR1 treatment on the phosphorylation state of 49 RTKs using a human phospho-RTK array, which revealed a significant decrease in the phosphorylation of multiple RTKs in both NCI-H1975 (Fig. 1a) and PC-9 (Supplementary Fig. 1a) cells. Consistent with our previous observation^15^, EGFR phosphorylation was not affected. We further tested various growth factors, including IGF-I and -II, insulin and platelet-derived growth factor (PDGF) in NCI-H1975 cells (Fig. 1b), as well as IGF-I and -II, insulin and HGF in PC-9 cells (Supplementary Fig. 1b), and verified that the siROR1 treatment effectively inhibited growth factor-induced phosphorylation of RTKs and AKT. These findings led us to hypothesize that the inhibitory effects on the signalling of multiple RTKs may be caused by impairment of the caveolae structure; RTKs are in part localized in caveolae^4^. Accordingly, CAV1 was knocked down in the NCI-H1975 and PC-9 cell lines (Fig. 1c and Supplementary Fig. 1c, respectively). We observed faithful recapitulation of the inhibitory effects of ROR1 knockdown, which suggested that ROR1 may be involved in the regulation of caveolae in NSCLC cells.

### Sustainment of CAV1 expression and caveola structure by ROR1

**Figure 2:**
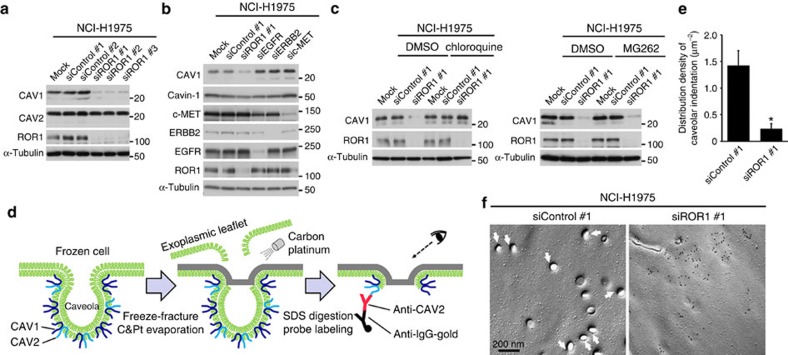
ROR1 inhibits the lysosomal degradation of CAV1 and sustains intact caveolae structures. (**a**) Decreased CAV1 but not of CAV2 expression with the use of three independent ROR1 siRNAs. See Supplementary Figs 2a–e and 3, which shows similar effects in other cell lines. (**b**) Reduced CAV1 expression by knocking down ROR1 but not EGFR, ERBB2 or MET in NCI-H1975 cells. Note that cavin-1 expression is not affected. (**c**) Rescue of CAV1 expression by treatment with a lysosome inhibitor (left panel) but not by treatment with a proteasome inhibitor (right panel) in NCI-H1975 cells knocked down for ROR1. (**d**) Schematic diagram of immunoelectron microscopy of SDS-treated freeze-fracture replica. (**e**) Decreased number of typical caveolae structures in the plasma membranes of the siROR1-treated NCI-H1975 cells were observed in the electron microscopic examination. More than 18 random plasma membrane areas of 17–50 μm^2^ were examined for both samples. (average±s.e.m.; Student’s *t*-test; *P<0.001.) (**f**) Representative results of the freeze-fracture immunoelectron microscopy. CAV2 labels made clusters, but the labelled membrane was either flat or superficially depressed in the siROR1-treated NCI-H1975 cells. By contrast, the CAV2 labelling in the siControl-treated NCI-H1975 cells showed intact caveolae with indentations measuring 50–75 nm in diameter. Also refer to Supplementary Fig. 4. Uncropped images of blots are shown in Supplementary Fig. 11.

We next investigated whether ROR1 was required for CAV1 expression. Western blot (WB) analysis revealed significantly decreased the expression of CAV1 but not CAV2 protein expression in NCI-H1975 cells treated with three independent small interfering RNAs (siRNAs) against ROR1 (Fig. 2a). Immunofluorescence staining analysis showed similar results (Supplementary Fig. 2a). In contrast to previous reports of the co-disappearance of CAV1 and CAV2 in other cell types^17^,^18^, CAV2 expression was preserved in NCI-H1975 cells knocked down for CAV1 (Supplementary Fig. 2b), suggesting the existence of cell type-specific dependency. In addition, immunofluorescence staining of IGF-IR was affected similarly by siROR1 and siCAV1 treatments (Supplementary Fig. 2c). We verified the specificity of siROR1 treatment by rescuing with siRNA-resistant wild-type ROR1 (Supplementary Fig. 2d). Quantitative reverse transcriptase-PCR (RT-PCR) analysis showed no change in CAV1 mRNA expression in the siROR1-treated NCI-H1975 cells (Supplementary Fig. 2e), suggesting the involvement of a posttranscriptional mechanism. The siROR1-induced reduction of CAV1 was confirmed in three additional NSCLC cell lines, NCI-H441, NCI-H358 and PC-9 cells (Supplementary Fig. 3a). siROR1 treatment also significantly reduced CAV1 in A431 (vulval epidermoid carcinoma) and HeLa (cervical cancer) cell lines, indicating the involvement of ROR1 in other types of cancer cells (Supplementary Fig. 3b). Consistent with previous reports^19^,^20^, exogenously overexpressed CAV1 exhibited a similar distribution in COS-7 cells, while siROR1 treatment markedly reduced both endogenous and exogenous CAV1 expression (Supplementary Fig. 3c,d). In contrast to ROR1 knockdown, treatment with siEGFR, siERBB2 or siMET did not affect CAV1 expression levels (Fig. 2b). Next, because the CAV1 protein is thought to be regulated by the endosome–lysosome system^5^, we investigated whether the observed siROR1-induced CAV1 reduction could be rescued by chloroquine, an inhibitor of lysosomal degradation. The results showed that chloroquine treatment clearly rescued CAV1 expression from the siROR1-induced reduction. By contrast, we observed no effect with the MG262 proteasome inhibitor (Fig. 2c).

We then analysed the caveolae structure using freeze-fracture replica electron microscopy^21^,^22^, which (in contrast to ultrathin sections) can be used to observe wide two-dimensional areas of the membrane, distinguishing shallow caveolae from undulations that occur in many other parts of the plasma membrane (Fig. 2d). We consequently found that siROR1 treatment resulted in a significantly smaller number of caveolae per unit membrane area compared with control siRNA treatment in NCI-H1975 (Fig. 2e). In addition, the ultrastructural localization of CAV2 in cells knocked down for ROR1 was examined using immunoelectron microscopy. In contrast to CAV2 labelling observed in the typical caveolae structure in control siRNA-treated cells, in the siROR1-treated cells, a majority of CAV2 labels were found in the membrane areas that showed no morphological differentiation or were only shallowly depressed in both NCI-H1975 (Fig. 2f) and A431 (Supplementary Fig. 4a). In addition, we employed freeze-etching electron microscopy to observe the cytoplasmic surface of the plasma membrane^23^ and verified that the siROR1-treated cells obviously lacked typical caveolae structures but retained clathrin-coated pits (Supplementary Fig. 4b). These findings clearly indicated that ROR1 is required to sustain CAV1 expression and the resultant caveolae formation.

**Figure 3:**
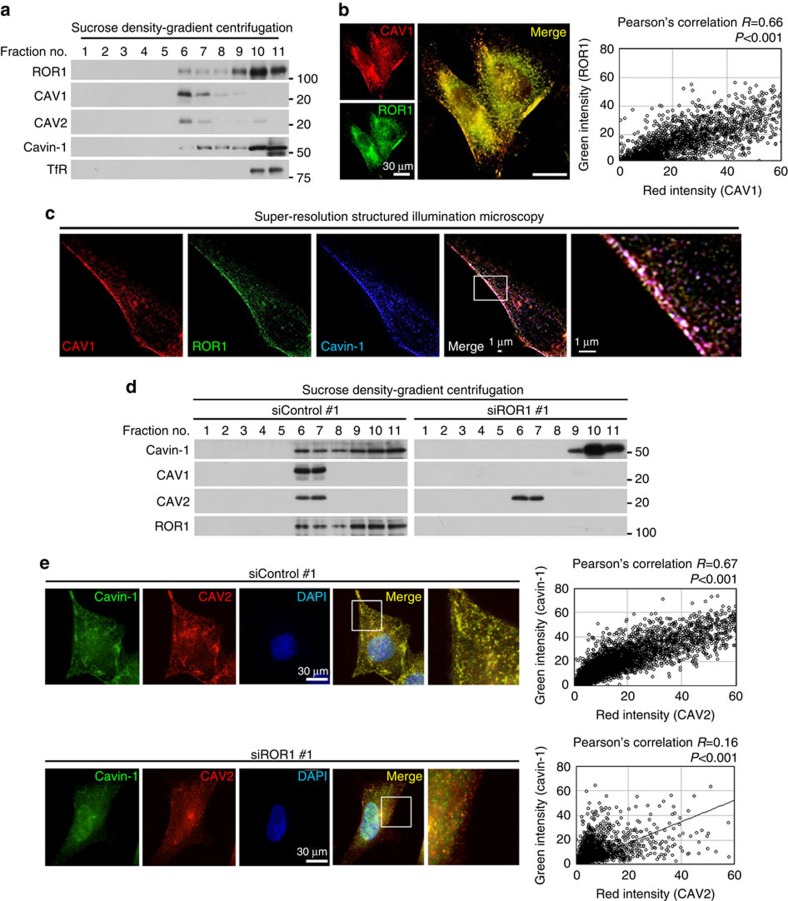
ROR1 colocalizes with CAV1 and cavin-1 and retains cavin-1 in DRM. (**a**) Sucrose density-gradient centrifugation confirmed the presence of ROR1 and cavin-1 in the DRM fractions containing CAV1 and CAV2 in the NCI-H1975 cells. (**b**) ROR1 and CAV1 colocalization shown by two-colour immunofluorescence staining in NCI-H1975 cells. Colocalization was quantified using ImageJ software. Also see Supplementary Fig. 5. (**c**) The colocalization of ROR1 with CAV1 and cavin-1 shown by three-colour immunofluorescence staining using super-resolution structured illumination microscopy in NCI-H1975 cells. (**d**) Sucrose density-gradient centrifugation showing the loss of CAV1 as well as marked changes of cavin-1 subcellular distribution in NCI-H1975 cells knocked down for ROR1. Also see Supplementary Fig. 6a. (**e**) Two-colour immunofluorescence staining showing markedly impaired colocalization between cavin-1 and CAV2 induced by ROR1 knockdown in NCI-H1975 cells. Colocalization was quantified using ImageJ software. Also see Supplementary Fig. 6b. Uncropped images of blots are shown in Supplementary Fig. 11.

We next employed sucrose density-gradient centrifugation to separate the detergent-resistant membrane (DRM), including caveolae, from the bulk cellular proteins^24^ and performed a WB using the recovered fractions. A proportion of ROR1 was found to reside in the DRM fractions, which contained the caveolae-specific proteins CAV1 and CAV2 (Fig. 3a). Two-colour immunofluorescence staining revealed the colocalization of the punctate signals of ROR1 with those of CAV1 (Fig. 3b and Supplementary Fig. 5a) and cavin-1 (Supplementary Fig. 5b). Immunofluorescence staining combined with the unroofing procedure^25^ was also employed, confirming colocalization of the punctate signals of ROR1 and CAV1 (Supplementary Fig. 5c). In addition, the colocalization of ROR1, CAV1 and cavin-1 was verified at a much higher resolution by three-colour immunofluorescence staining using super-resolution structured illumination microscopy (SIM)^26^ (Fig. 3c). The siROR1 treatment resulted in a significant decrease in the CAV1 expression, as well as a near-complete loss of cavin-1, specifically in the Triton X-insoluble fraction (Supplementary Fig. 6a). We further verified this finding using sucrose density-gradient centrifugation and observed that ROR1 knockdown resulted in not only a marked decrease in the total CAV1 expression but also a significant reduction of cavin-1 in the DRM fraction containing CAV2 (Fig. 3d). Consistently, punctate cavin-1 signals at the plasma membrane were significantly decreased (Supplementary Fig. 6b), and double immunofluorescence staining showed a markedly diminished colocalization between cavin-1 and CAV2 in the NCI-H1975 cells knocked down for ROR1 (Fig. 3e). The present findings indicate that the ROR1 residing in the caveolae sustains CAV1 expression by preventing its lysosomal degradation, which consequently enables caveolae to be formed in the plasma membranes of NSCLC cells.

### ROR1 kinase-independent sustainment of CAV1 expression

**Figure 4:**
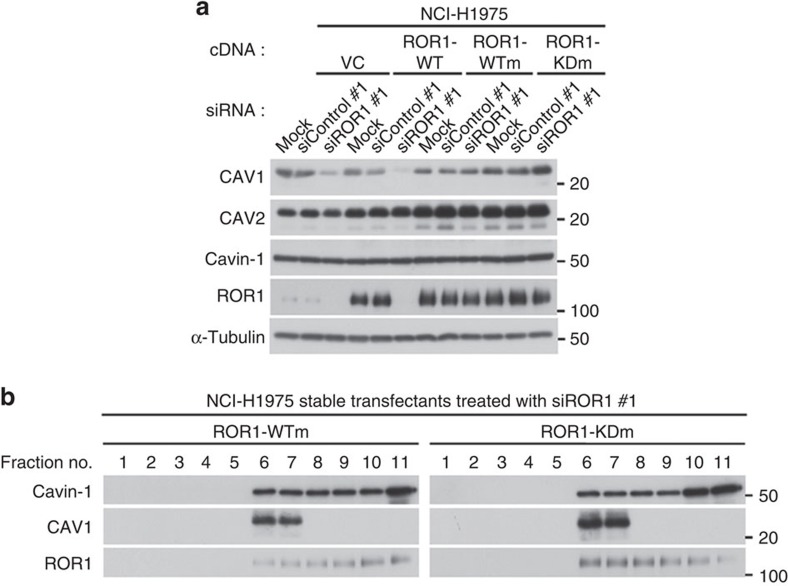
ROR1 kinase activity is not required to sustain CAV1 expression. (**a**) Sustainment of CAV1 expression in the presence of siROR1 by introduction of both siRNA-resistant, wild-type (wt) and kinase-dead ROR1 in the NCI-H1975 cells. Also see Supplementary Fig. 7. (**b**) Sucrose density-gradient assay showing that ROR1 kinase activity is dispensable for the retention of cavin-1 in DRM fractions. NCI-H1975 cells stably transfected with either wt or kinase-dead forms of siRNA-resistant ROR1 were subjected to siROR1 treatment in this analysis. Uncropped images of blots are shown in Supplementary Fig. 11.

We then investigated whether ROR1 kinase activity is required to sustain CAV1 expression and cavin-1 compartmentalization into the DRM. We found that reconstitution with the siRNA-resistant kinase-dead ROR1 could rescue CAV1 expression from siROR1 treatment as efficiently as siRNA-resistant wild-type ROR1 (Fig. 4a). Sucrose density-gradient centrifugation followed by WB analysis further verified that the sustainment of CAV1 in the DRM did not require ROR1 kinase activity (Fig. 4b). We also noted that cavin-1 was also retained in the DRM fraction due to the presence of kinase-dead ROR1. The present study revealed impaired phosphorylation of multiple RTKs in NSCLC cells knocked down for ROR1. We also observed that IGF-I-stimulated IGF-IR phosphorylation was maintained by the presence of kinase-dead ROR1 (Supplementary Fig. 7a). Although ROR1 phosphorylates SRC^15^, treatments with siSRC or SRC kinase inhibitors (dasatinib and SKI-I) did not alter CAV1 expression (Supplementary Fig. 7b). The replacement of all five potential tyrosine phosphorylation sites of cavin-1 did not affect cavin-1 subcellular compartmentalization (Supplementary Fig. 7c). These findings indicate that ROR1 possesses a kinase activity-independent function that is required to compartmentalize cavin-1 into the DRM and to sustain CAV1 expression in NSCLC cells.

### Identification of cavin-1 and CAV1 as ROR1-binding proteins

**Figure 5:**
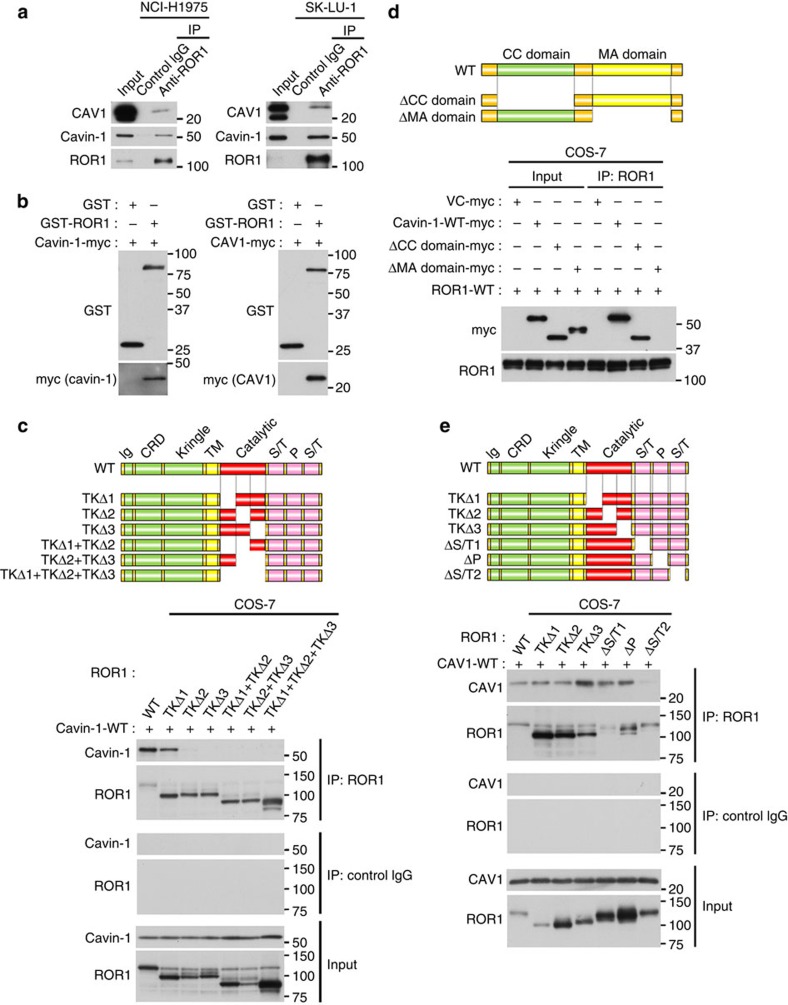
ROR1 interacts with both cavin-1 and CAV1 at two respective binding regions. (**a**) IP–WB analysis using octylglucoside as a detergent showed association of ROR1 with cavin-1 and CAV1. See Supplementary Fig. 8a. Also see Supplementary Fig. 8b for pull-down assay showing their mutual associations. (**b**) Pull-down assay using purified proteins of ROR1, cavin-1, and CAV1 showing physical associations of ROR1 with cavin-1 and CAV1. (**c**) Identification of C-terminal two-thirds of the ROR1 kinase domain as the cavin-1 binding region by IP-WB analysis using various ROR1 deletion mutants. Also see Supplementary Fig. 8c–f. (**d**) Identification of the membrane association domain of cavin-1 as its ROR1-binding region by IP–WB analysis using cavin-1 deletion mutants. Also see Supplementary Fig. 8g. (**e**) Mapping of the CAV1 binding region to the C-terminal serine/threonine-rich domain by IP–WB analysis using various ROR1 deletion mutants. Uncropped images of blots are shown in Supplementary Fig. 11.

Evidence indicates that the binding of cavin-1 with CAV1 at the plasma membrane protects CAV1 from lysosome-dependent degradation and leads to caveolae formation^5^. To obtain a more in-depth insight into how ROR1 participates in this process, we first investigated whether ROR1 binds with cavin-1 and/or CAV1. We performed an immunoprecipitation (IP)–WB analysis using octylglucoside as a detergent. Octylglucoside preserves protein–protein interactions but efficiently solubilizes DRM^6^. We found that ROR1 interacted with both cavin-1 and CAV1 in the NCI-H1975 and SK-LU-1 cells (Fig. 5a), as well as in the NCI-H441 and PC-9 cells (Supplementary Fig. 8a). Mutual interactions of each protein pair in ROR1, cavin-1 and CAV1 were also demonstrated by pull-down assays using the respective purified glutathione *S*-transferase (GST)-tagged proteins and cell lysates of the NCI-H1975 cells (Supplementary Fig. 8b). In addition, their interactions were further confirmed by pull-down assay using a GST-tagged purified ROR1, as well as a myc-tagged purified cavin-1 and CAV1 (Fig. 5b).

**Figure 6:**
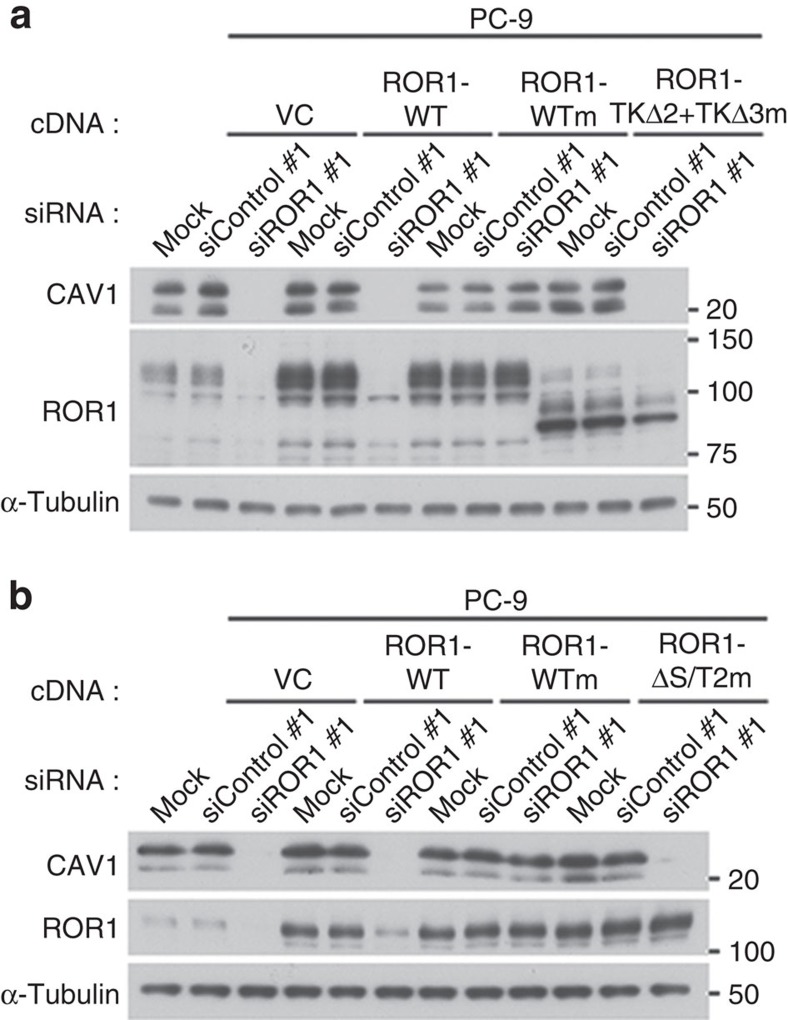
ROR1 sustains CAV1 expression through cavin-1 and CAV1 binding. (**a**) Indispensable involvement of the cavin-1-binding region of ROR1 in sustaining CAV1 expression shown in PC-9 cells reconstituted with ROR1 lacking the cavin-1-binding region. (**b**) Indispensable involvement of the CAV1 binding region of ROR1 for sustained CAV1 expression shown in PC-9 cells reconstituted with ROR1 lacking the CAV1-binding region. Uncropped images of blots are shown in Supplementary Fig. 11.

We next determined their respective binding regions using various deletion mutants. The IP–WB analysis showed that the interaction between ROR1 and cavin-1 required the intracellular domain of ROR1 but not ROR1 kinase activity (Supplementary Fig. 8c,d). Although the most C-terminal portion of the ROR1 intracellular domain contains two serine/threonine-rich domains and a proline-rich domain^27^, all domains were also dispensable for the interaction between ROR1 and cavin-1 (Supplementary Fig. 8e). By contrast, the C-terminal two-thirds of the ROR1 kinase domain were found to be necessary for the binding between ROR1 and cavin-1 (Fig. 5c). Conversely, the two-thirds of the ROR1 kinase domain alone could associate with cavin-1 (Supplementary Fig. 8f). Cavin-1 is known to harbour two functional domains, namely coiled-coil and membrane association domains^1^, and a deletion mutant of cavin-1 lacking the latter but not the former domain failed to interact with ROR1, as demonstrated using IP–WB analysis (Fig. 5d). A pull-down assay using GST-tagged cavin-1 and NCI-H1975 cell lysate further indicated that the membrane association domain was the ROR1-binding region of cavin-1 (Supplementary Fig. 8g). We also used various deletion mutants to investigate which portion of ROR1 was required for its interaction with CAV1; we identified the most C-terminal serine/threonine-rich domain of ROR1 as a region for their interaction (Fig. 5e). Notably, an siRNA-resistant deletion mutant of ROR1 lacking either the cavin-1- or CAV1-binding region failed to rescue CAV1 expression abrogated by siROR1 treatment, which clearly depleted endogenous ROR1. These results indicate the functional importance of both ROR1–cavin-1 and ROR1–CAV1 interactions (Fig. 6a,b).

**Figure 7:**
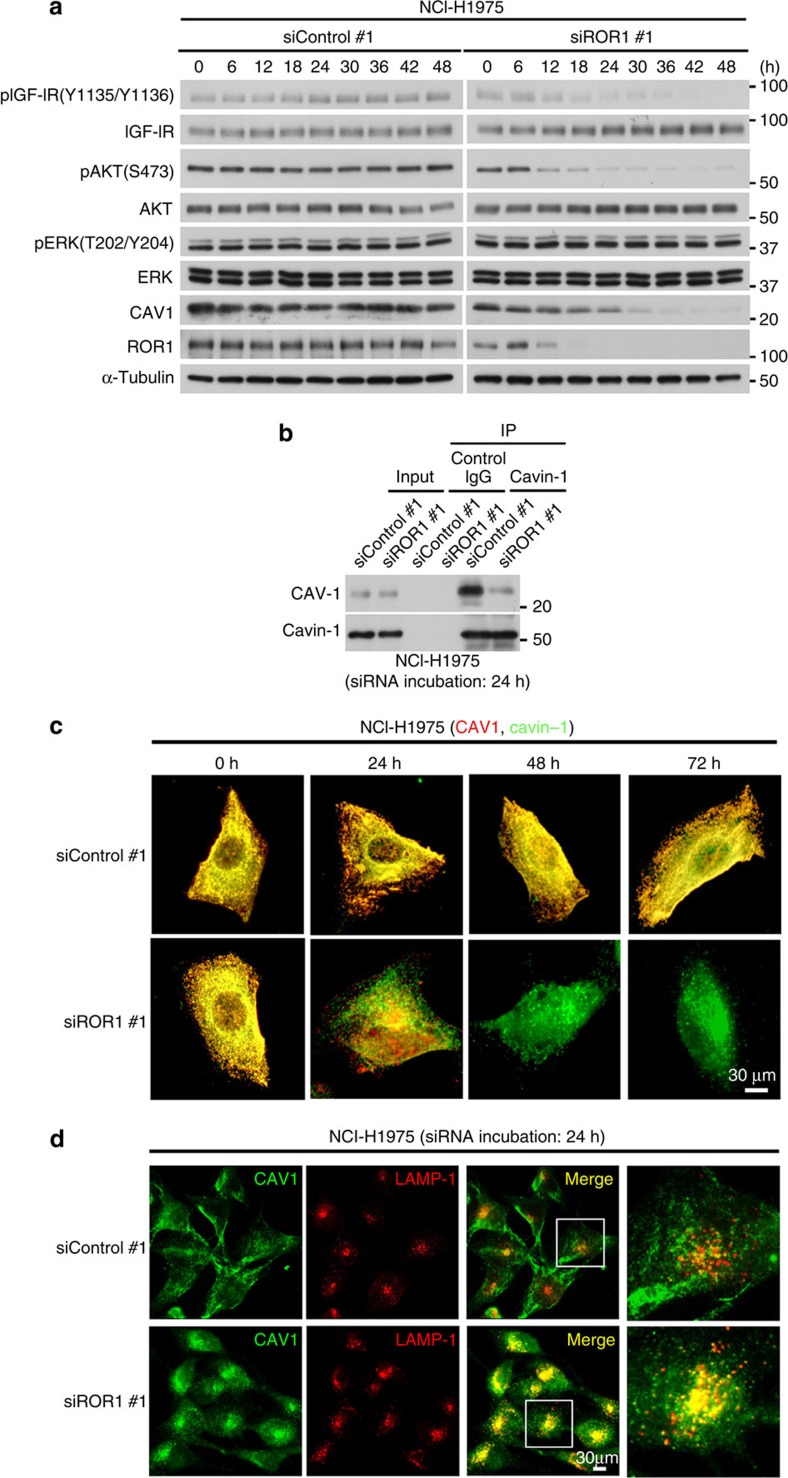
ROR1 is required for the prevention of CAV1 being routed to the lysosome. (**a**) WB analysis showing a gradual decrease and an easily detectable retention of CAV1 expression 24 h after siROR1 transfection in NCI-H1975 cells. (**b**) IP–WB analysis showing the impaired association between cavin-1 and CAV1 in siROR1-treated NCI-H1975 cells. Cell lysates harvested 24 h after siROR1 transfection, when the siROR1-induced reduction of CAV1 expression was not yet obviously elicited. (**c**) Two-colour immunofluorescence analysis showing a significant loss of colocalization between CAV1 and cavin-1 24 h after siROR1 treatment. (**d**) Two-colour immunofluorescence analysis showing significant colocalization of CAV1 with LAMP-1 24 h after siROR1 treatment. Uncropped images of blots are shown in Supplementary Fig. 11.

CAV1 expression gradually decreased but remained readily detectable 24 h after siROR1 transfection, while the phosphorylation of AKT and IGF-IR was already significantly reduced at this time point (Fig. 7a). Therefore, we investigated whether ROR1 knockdown affected the interaction between cavin-1 and CAV1 by taking advantage of the delayed reduction of CAV1 after the transfection of siROR1. A significant decrease in the association between cavin-1 and CAV1 was observed in NCI-H1975 cells 24 h after siROR1 transfection (Fig. 7b), and consistently, two-colour immunofluorescence analysis revealed a significant loss of CAV1 and cavin-1 colocalization (Fig. 7c). Moreover, we found that the subcellular localization of CAV1 was markedly altered at that time point, leading to significant colocalization of CAV1 with LAMP-1, an endosome/lysosome marker (Fig. 7d). Altogether, the present findings indicate that ROR1 possesses a novel function as an indispensable scaffold protein of cavin-1 and CAV1, which in turn prevents lysosomal CAV1 degradation and sustains CAV1 expression and caveolae formation.

### ROR1 sustains EGFR–TKI resistance-conferring bypass pathways

**Figure 8:**
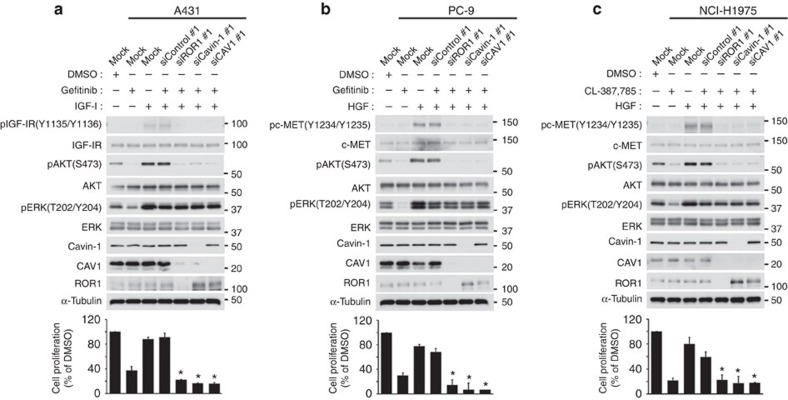
ROR1 inhibition overcomes bypass signalling-mediated EGFR–TKI resistance. (**a**) IGF-I-induced, IGF-IR-mediated bypass-signalling inhibition and the consequent gefitinib resistance by siROR1, sicavin-1 or siCAV1 treatment in the A431 vulval epidermoid carcinoma cell line. Also see Supplementary Fig. 9. (**b**) HGF-induced, MET-mediated, bypass-signalling inhibition and consequent gefitinib resistance following knockdown of ROR1, cavin-1 or CAV1 in the PC-9 lung adenocarcinoma cell line. (**c**) Inhibition of HGF-induced bypass signalling and consequent CL-387, 785 resistance by knocking down of ROR1, cavin-1 or CAV1 in the NCI-H1975 lung adenocarcinoma cell line. All data are represented as the mean±s.e.m. (*n*=3). *P<0.001 versus siControl, as determined by Student’s *t*-test. Uncropped images of blots are shown in Supplementary Fig. 11.

We observed that cell proliferation was significantly inhibited in the NCI-H1975 and PC-9 lung adenocarcinoma cell lines by not only siROR1 treatment but also the knockdown of either cavin-1 or CAV1 (Supplementary Fig. 9a), suggesting that the scaffold function of ROR1 for caveolae formation may be crucially involved in ROR1-mediated TTF-1/NKX2-1 lineage-survival signalling. The inevitable acquisition of EGFR–TKI resistance due to bypass signalling through other non-targeted RTKs is currently a major clinical obstacle^10^,^11^. Based on the present findings, we therefore investigated the possibility that the inhibition of ROR1, cavin-1 or CAV1 could overcome such bypass signalling-induced EGFR–TKI resistance. The A431 vulval epidermoid carcinoma cell line overexpressing EGFR exhibited a significant response to an EGFR–TKI (gefitinib) but became resistant when co-treated with IGF-I^13^. However, siROR1 treatment overcame the IGF-I-elicited increase in the phosphorylation of IGF-IR and AKT and resulted in growth inhibition (Fig. 8a). In addition, cavin-1 or CAV1 knockdown was effective for overcoming IGF-I-mediated gefitinib resistance. Cavin-1 or CAV1 knockdown also resulted in the efficient reversal of HGF-mediated, MET-transduced gefitinib resistance in PC-9 cells, as observed with siROR1 treatment (Fig. 8b)^15^. Similarly, HGF-conferred resistance against CL-387, 785, an irreversible EGFR–TKI inhibitor^28^,^29^,^30^, was reverted by treatment with siROR1, sicavin-1 or siCAV1 in NCI-H1975 cells carrying a gefitinib resistance-conferring T790M EGFR mutation (Fig. 8c). These findings suggest that inhibiting ROR1 may be an attractive approach to treating human cancers including those exhibiting EGFR–TKI resistance due to bypass signalling through other non-targeted RTKs such as MET and IGF-IR.

## Discussion

**Figure 9:**
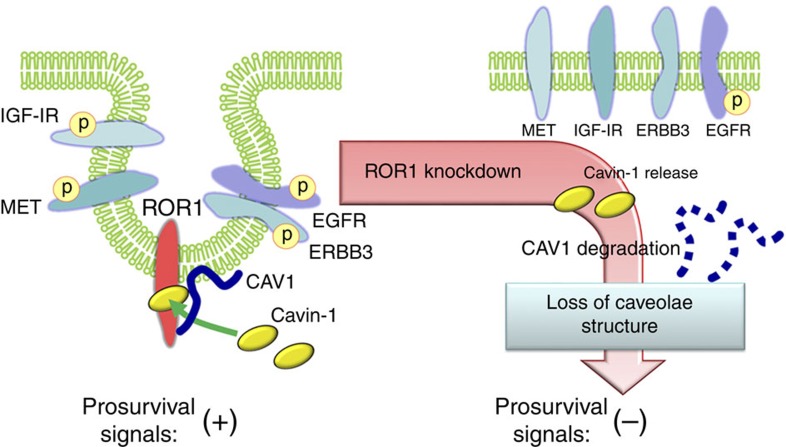
Schematic diagram of proposed model showing how ROR1 sustains caveolae formation. ROR1 facilitates the interaction of cavin-1 and CAV1 at the plasma membrane in a kinase activity-independent manner, which in turn sustains caveolae formation and prosurvival signalling towards AKT through multiple RTKs via its scaffold function for cavin-1 and CAV1 in human cancer cells. Our results also provide mechanistic insight into how ROR1 inhibition can overcome EGFR–TKI resistance caused by bypass signalling via diverse RTKs.

This study revealed an unanticipated function of the ROR1 RTK in NSCLCs. We found that ROR1 plays the role of a scaffold protein for cavin-1 and CAV1, facilitating their associations at the plasma membrane. This kinase-independent function of ROR1 maintains CAV1 expression by preventing its lysosome-dependent degradation, as well as consequential caveolae formation, which in turn sustains prosurvival signalling towards AKT from multiple RTKs such as EGFR, MET and IGF-IR (Fig. 9). The proposed model is in agreement with the well-established importance of the association between cavin-1 and CAV1 in caveolae formation^1^,^2^,^3^,^6^. CAV1 at the Golgi level does not recruit cavin-1 to form a complex, and the co-recruitment of CAV1 and cavin-1 to the plasma membrane is a prerequisite for their association and for caveolae formation. Cavin-1 and CAV1 were previously shown in fluorescence resonance energy transfer (FRET) experiments to be in close proximity to each other at the plasma membrane^5^. In this regard, the present findings provide mechanistic insight into how the two essential components of caveolae (cavin-1 and CAV1) are co-recruited to the plasma membrane with the aid of transmembrane ROR1.

This study provides clear evidence that ROR1 is required to sustain caveolae structure in multiple NSCLC cell lines, two other cancer cell lines and COS-7 cells. It is also conceivable that ROR1 is involved in caveolae formation in various fetal tissues including the lung, which abundantly express ROR1 (refs 27, 31). However, we think that it is unlikely for ROR1 to be invariably required for caveolae formation, considering its negligible expression or absence of expression in normal human adult tissues^27^,^31^. In this regard, low-affinity interactions of CAV1 and cavin-1 with phosphatidylserine have been suggested to be involved in caveolae formation in adipose cells, which do not express ROR1 (refs 5, 32). Regarding the existence of marked tissue-type specificities in CAV1 expression and caveolae density, distinct molecular mechanisms may be involved in the sustainment of caveolae structures in a given cell state and lineage, which would potentially affect the mode of caveolae function.

We observed that ROR1 knockdown resulted in the decreased phosphorylation of AKT but not ERK in NSCLC cells stimulated with various growth factors including EGF, HGF, IGF-I, IGF-II, insulin and PDGF. It is also of note that this specific signalling impairment was recapitulated by knocking down cavin-1 or CAV1. These findings indicate that a certain set of signalling downstream of RTKs may require intact caveolae structure. We note that such differential dependence on intact caveolae was previously reported in insulin-elicited signalling in rodents but not human adipocytes^4^,^33^, suggesting the possible existence of species- and tissue-type-specific differences in terms of the dependence on intact caveolae structures. Taken together, the present findings strongly indicate that ROR1 plays a crucial role as a scaffold protein in the sustainment of caveolae structure and prosurvival signalling of multiple RTKs in NSCLCs. We think that the novel scaffold function of ROR1 discovered in this study may primarily sustain prosurvival signalling, thereby enabling other previously described ROR1 functions to become engaged in response to EGF stimulation^15^.

This study clearly shows that the impairment of caveolae formation by cavin-1 or CAV1 knockdown leads to significant growth inhibition in NSCLC cell lines, as also observed after siROR1 treatment. John Minna and colleagues previously reported siCAV1-induced growth inhibition in NSCLC cell lines^9^; however, the underlying mechanism remained rather ambiguous. CAV1 and cavin-1 themselves are unlikely to be suitable molecular targets for NSCLC treatment due to their crucial physiological functions in various organs even at low-expression levels^17^,^34^,^35^,^36^,^37^,^38^,^39^,^40^. In this regard, it must be noted that ROR1 is considered to be an onco-embryonic antigen with tumour-specific expression in adults^27^,^31^. Moreover, ROR1 appears to possess a distinctive attractiveness as a molecular target for the treatment of NSCLCs, especially lung adenocarcinoma. Diverse RTKs are widely known to be involved in EGFR–TKI resistance via bypass signalling^12^,^13^,^14^,^41^. This involvement makes it difficult to predict which RTK should be targeted to prevent the expansion of resistant clones in each patient. This study clearly shows that inhibition of prosurvival signalling arising from a broad spectrum of RTKs can be attained through the disruption of caveolae structures by knocking down ROR1, cavin-1 or CAV1; the scaffold function of ROR1 is therefore an attractive target for overcoming EGFR–TKI resistance due to bypass signalling. Finally, we note that ROR1 has been suggested to play a role in the development of not only lung cancers^15^,^42^,^43^ but also various other human malignancies including cancers of the breast^44^, pancreas^43^, stomach^42^, colon^43^, ovary^45^ and skin^27^, as well as acute and chronic leukaemias^31^,^46^,^47^,^48^.

In summary, the ROR1 RTK possesses an unanticipated function as a scaffold protein of cavin-1 and CAV1 in a kinase activity-independent manner, which in turn sustains caveolae formation and prosurvival signalling onto AKT through multiple RTKs in NSCLCs. Novel therapeutic strategies to inhibit the scaffold function of ROR1 and thereby attack the cancer’s ‘Achilles heel’ may prove effective against this devastating cancer.

## Methods

### Cell lines

NCI-H1975, NCI-H441, NCI-H358, A431 and HeLa cells were purchased from the American Type Culture Collection. PC-9 cells were purchased from the RIKEN Cell Bank. SK-LU-1 was a gift from the late Dr Lloyd J. Old (Memorial Sloan Kettering Cancer Center). The cells were maintained in RPMI-1640 with 10% fetal bovine serum. All cell lines were authenticated by short tandem repeat DNA profiling and were free of mycoplasma contamination.

### Reagents

Gefitinib and dasatinib were purchased from Biaffin GmbH & Co KG (Kassel, Germany) and Selleckchem (Houston, TX), respectively. CL-387, 785 and SKI-I were purchased from Calbiochem (San Diego, CA). Recombinant HGF, IGF-I and IGF-II were obtained from PeproTech (Rocky Hill, NJ). Recombinant insulin and PDGF were obtained from Sigma (St Louis, MO) and Cell Signaling Technology, respectively. Lysosomal inhibitor (Chloroquine) and proteosomal inhibitor (MG262) were purchased from Sigma. Anti-ROR1 (#4102/rabbit, 1:50), anti-IGF-IR (#3027/rabbit, 1:50), anti-PDGFR (#3164/rabbit, 1:50), anti-c-MET (#3127/clone#25H2/mouse, 1:50), anti-ERBB2 (#2165/clone#29D8/rabbit, 1:200), anti-AKT (#4691/clone#C67E7/rabbit, 1:500), anti-ERK (#9102/rabbit, 1:500), anti-c-Src (#2109/clone#36D10/rabbit, 1:1,000), anti-S6K (#9202/rabbit, 1:200), anti-phospho-IGF-IR (Y1135/Y1136) (#3024/clone#19H7/rabbit, 1:50), anti-phospho-InsulinR (Y1150/Y1151) (#3024/clone#19H7/rabbit, 1:50), anti-phospho-PDGFR (Y754) (#2992/clone#23B2/rabbit, 1:50), anti-phospho-c-MET (Y1234/Y1235) (#3126/rabbit, 1:50), anti-phospho-AKT (S473) (#9271/rabbit, 1:500) and anti-phospho-ERK1/2 (T202/Y204) (#4377/clone#197G2/rabbit, 1:500), anti-phospho-c-Src (Y416) (#2101/rabbit, 1:200) and anti-phospho-S6K (T389) (#9205/rabbit, 1:50) were purchased from Cell Signaling Technology, while anti-α-tubulin (T9026/clone#DM1A/mouse, 1:40,000) from Sigma, anti-c-myc (sc-40/clone#9E10/mouse, 1:200) from SantaCruz (Santa Cruz, CA), anti-Transferrin receptor (13–6,800/clone#H68.4/mouse, 1:50) from Invitrogen (Carlsbad, CA), anti-cavin-1 (A301-271 A/rabbit, 1:200) from Bethyl Laboratories (Montgomery, TX), anti-GST (M071-3/clone#3B2/mouse, 1:1,000) from MBL (Nagoya), anti-ROR1 (IP-specific, AF2000/goat, 1:250) from R&D systems (Minneapolis, MN), anti-InsulinR (ab69508/clone#C18C4/mouse, 1:200), anti-ROR1 (IF-specific, ab111174/goat, 1:200), anti-CAV2 (IF-specific, ab75865/rabbit, 1:20), anti-CAV1 (ab18199/rabbit, 1:20), anti-LAMP-1 (ab24170/rabbit, 1:20), and anti-cavin-1 (IF-specific, ab135655/rabbit, 1:20) from Abcam (Cambridge, MA), anti-CAV1 (610407/clone#2297/mouse, 1:500 for WB and 1:20 for immunofluorescence microscopy), anti-CAV2 (610685/clone#65/mouse) and anti-EGFR (610017/clone#13/mouse, 1:500) from BD Bioscience (Bedford, MA) and anti-mouse IgG (#7076, 1:1,000) and anti-rabbit IgG (#7074, 1:1,000) from Cell Signaling Technology. The primer sequences used for *in vitro* mutagenesis and quantitative RT-PCR are now provided as Supplementary Information, as are the siRNA sequences. siControl #1 (AllStars Negative Control siRNA) and siControl #2 (Negative Control) were obtained from QIAGEN and Sigma, respectively.

### Constructs

Constructions of pCMVpuro-ROR1 and pCMVpuro-ROR1-KD as well as pIRESpuro2-ROR1-myc and its derivatives (pIRESpuro2-ROR1-ΔN-myc and pIRESpuro2-ROR1-ΔC-myc) were previously reported^15^. Based on the pCMVpuro-ROR1 vector, pCMVpuro-ROR1-TKΔ1 (Δ473–564), pCMVpuro-ROR1-TKΔ2 (Δ564–655), pCMVpuro-ROR1-TKΔ3 (Δ655–746), pCMVpuro-ROR1-TKΔ1+TKΔ2 (Δ473–655), pCMVpuro-ROR1-TKΔ2+TKΔ3 (Δ564–746), pCMVpuro-ROR1-TKΔ1+TKΔ2+TKΔ3 (Δ473–746), pCMVpuro-ROR1-ΔST1 (Δ748–782), pCMVpuro-ROR1-ΔP (Δ784–861), pCMVpuro-ROR1-ΔST2 (Δ853–876), pCMVpuro-ROR1-ΔP+ΔST2 (Δ784–876) and pCMVpuro-ROR1-ΔST1+ΔP+ΔST2 (Δ748–876) were prepared by *in vitro* mutagenesis using KOD-Plus-DNA polymerase (TOYOBO) according to the manufacturer's instructions. pIRESpuro2-ROR1-TK1+TK2+TK3-myc and pIRESpuro2-ROR1-TK2+TK3-myc were constructed by the *in vitro* mutagenesis of pIRESpuro2-ROR1-myc using a KOD-Plus-DNA polymerase. pCMVpuro-ROR1-WTm, pCMVpuro-ROR1-KDm, pCMVpuro-ROR1-ΔTK2+ΔTK3m and pCMVpuro-ROR1-ΔS/T2m carrying multiple silent mutations at the binding site of siROR1#1 were constructed by *in vitro* mutagenesis using KOD-Plus-DNA polymerase (TOYOBO) and the oligonucleotide primer 5′-CAACAGTGGACAGAGTTCCAG-3′ (mutated residues are underlined).

In addition, Myc-tagged full-length human cavin-1 and CAV1 cDNA were purchased from OriGene Technologies and inserted into a pCMVpuro vector (pCMVpuro-cavin-1-WT-myc and pCMVpuro-CAV1-WT-myc). pCMVpuro-cavin-1-ΔCCD-myc (Δ46–166) and pCMVpuro-cavin-1-ΔMAD-myc (Δ217–298) were then prepared by *in vitro* mutagenesis using a KOD-Plus-DNA polymerase. pCMVpuro-cavin-1-WTm-myc and pCMVpuro-cavin-1–5Fm-myc carrying multiple silent mutations at the binding site of sicavin-1#1 were constructed by *in vitro* mutagenesis using KOD-Plus-DNA polymerase (TOYOBO) and the oligonucleotide primer 5′-TCAAAAACAGCAGTATTC-3′ (mutated residues are underlined). The entire open reading frames of the resultant constructs were thoroughly sequenced.

### RNA interference

Cells were seeded at 5.0 × 10^4^ cells per well in six-well plates for immunofluorescence microscopy; at 1.0 × 10^5^ per well in six-well plates for WB, quantitative RT-PCR and electron and immunoelectron microscopy; and at 1 × 10^6^ in 10 cm dishes for IP-WB, phospho-RTK array, cell fractionation and sucrose density-gradient centrifugation analyses. On the next day, the cells were transfected with siRNAs (each at 20 nM) using Lipofectamine RNAiMAX (Invitrogen), according to the manufacturer's instructions. The cells were harvested for analyses 72 h after transfection. In the IP–WB analysis of the interaction between cavin-1 and CAV1 in siROR1-treated cells, the cells were harvested 24 h after siROR1 or siControl transfection. In the time course analysis, the cells were harvested or fixed at indicated time points after siROR1 or siControl transfection for WB analysis or immunofluorescence.

### WB and IP–WB analyses

WB and IP–WB analyses were performed according to standard procedures using Immobilon-P filters (Millipore) and an enhanced chemiluminescence system (GE Healthcare). To analyse the physical interactions between ROR1 and cavin-1 or CAV1, as well as those between cavin-1 and CAV1, whole-cell lysates of NSCLC cell lines were solubilized in octylglucoside buffer (60 mM octylglucoside, 150 mM NaCl and 50 mM EDTA) and immunoprecipitated with anti-ROR1, anti-cavin-1 or non-specific IgG antibodies. For determining the interacting region of ROR1 with either cavin-1 or CAV1, pCMVpuro-cavin-1-WT-myc or pCMVpuro-CAV1-WT-myc were transfected into COS-7 together with various ROR1 expression constructs. Similarly, pCMVpuro-ROR1-WT was transfected together with expression constructs of either the wild-type or the deletion mutant of cavin-1. The cells were harvested 24 h after transfection with the NP-40 lysis buffer containing 20 mM Tris-HCl (pH 8.0), 137 mM NaCl, 2 mM EDTA, 1% NP-40, 10% Glycerol and 1 mM Na_3_VO_4_ and a complete EDTA-free protease inhibitor mixture (Roche).

### Cell proliferation assay

We seeded 5.0 × 10^4^ cells per well in six-well plates 1 day before the siRNA treatment. Cell proliferation was measured 5 days after transfection by colorimetric assay using Cell Counting Kit-8 (Dojindo Laboratories).

### Phospho-RTK array analysis

A human Phospho-RTK Array kit containing duplicate validated controls and capture antibodies specific for 49 RTKs (R&D Systems) was used to simultaneously detect the relative tyrosine phosphorylation levels. A total of 1 × 10^6^ cells were seeded in 10 cm dishes 1 day before transfection with siControl, siROR1, or siCAV1 and were harvested 3 days after transfection. An RTK array analysis was performed according to the manufacturer’s protocol. In brief, the array membranes were blocked, incubated with each cell lysate overnight at 4 °C, washed, and incubated with anti-phosphotyrosine-horseradish peroxidase (HRP) for 2 h at room temperature, washed again, and developed with ECL western blotting detection reagents (GE Healthcare). The RTK spots were visualized with X-ray films (Fuji Photo Film). The average pixel densities of duplicate spots were determined using the ImageJ software (http://imagej.nih.gov/ij/).

### Cell fractionation assay

A total of 1 × 10^6^ cells were seeded in 10 cm dishes, transfected with siRNAs 24 h later, and harvested 3 days after transfection. The cytosolic fraction was extracted using a ProteoExtract Subcellular Proteome Extraction kit (Calbiochem) and subjected to Triton X-100 solubilization with TNE buffer (25 mM Tris-HCl (pH 7.5), 150 mM NaCl, 5 mM EDTA and protease inhibitors) containing 1% Triton X-100. The insoluble materials were then solubilized with 250 μl TNE buffer containing 1% SDS. Equal amounts of protein from each fraction were analysed by immunoblotting.

### Sucrose gradient centrifugation

A total of 1 × 10^6^ cells in 10 cm dishes were transfected with the indicated siRNAs and lysed with 1 ml of lysis buffer consisting of 1% triton X-100 and TN buffer (25 mM Tris-HCl (pH 7.5), 150 mM NaCl, 0.1 mM phenylmethylsulfonyl fluoride, 1 mM NaHSO_3_, 2 mM CaCl_2_ and 5 mM MgCl_2_). The lysates were transferred into centrifuge tubes (Ultra Clear, Beckman), stored on ice for 30 min, and then mixed with 1 ml of 80% sucrose in TN buffer. Total 5.5 ml of 30% sucrose and 3.5 ml of 5% sucrose solutions were subsequently layered onto the lysate in 40% sucrose and then centrifuged in a SW41Ti rotor (Beckman) at 200,000*g* at 4 °C for 22 h. Then, 1-ml fractions were collected from the top of the gradient with designations from number 1 through 11. Light-scattering, detergent-insoluble membrane fractions were located at the 5 and 30% sucrose interfaces. The proteins were precipitated in cold acetone, dissolved in SDS sample buffer and analysed by WB.

### Lysosomal inhibitor treatment in cells knocked down for ROR1

A total of 1 × 10^5^ cells per well were seeded in six-well plates and transfected 24 h later with 20 nM siControl or siROR1. One day after transfection, the lysosomal inhibitor chloroquine (100 μM), the proteosomal inhibitor MG262 (10 nM) or dimethylsulphoxide (DMSO) were added at the indicated concentrations. The cells were harvested for WB analysis after 48 h continuous exposure to the inhibitors.

### Clarification of ROR1–RNA interference effects

Stable transfectants expressing siRNA-resistant forms of wild-type (wt)-ROR1 or kinase-dead ROR1, were generated by introducing the respective plasmids (pCMVpuro-ROR1-WTm or pCMVpuro-ROR1-KDm) using FuGENE6 (Promega), followed by puromycin selection. The resultant stable clones were then seeded at 1 × 10^5^ cells per well in six-well plates for WB analysis and at 1 × 10^6^ cells in 10-cm dishes for sucrose gradient centrifugation, introduced with siControl or siROR1 on the next day, and harvested 3 days after siRNA transfection.

For the experiments on IGF-I-induced IGF-IR phosphorylation shown in Supplementary Fig. 7a, 1 × 10^5^ cells per well in six-well plates stably expressing VC or wt-ROR1 (pCMVpuro-ROR1-WT) or an siRNA-resistant form of wt-ROR1 (pCMVpuro-ROR1-WTm) or that of kinase-dead ROR1 (pCMVpuro-ROR1-KDm) were transfected with siROR1 or siControl. After 48 h of incubation, the cells were serum-starved for 24 h then treated with 50 ng ml^−1^ of IGF-I for 30 min and harvested for WB analysis.

For the rescue experiments of CAV1 expression, 1.0 × 10^6^ cells were first transfected in 10-cm dishes with pCMVpuro-VC, pCMVpuro-ROR1-WT, pCMVpuro-ROR1-WTm, pCMVpuro-ROR1-ΔTK2+ΔTK3m or pCMVpuro-ROR1-ΔS/T2m, followed by puromycin selection (1.5 μg ml^−1^) for 3 days. The resultant bulk transfectants were then re-seeded at 1 × 10^5^ cells in six-well plates and further introduced with siControl or siROR1 the next day. The cells were harvested for WB analysis 3 days after siRNA transfection.

### Clarification of the effects of cavin-1 RNA interference

Stable transfectants expressing siRNA-resistant forms of wt-cavin-1 or 5F-cavin-1 were generated by introducing the respective plasmids (pCMVpuro-cavin-1-WTm-myc and pCMVpuro-cavin-1–5Fm-myc) using FuGENE6 (Promega), followed by puromycin selection. The resultant stable clones were then seeded at 1 × 10^6^ cells in 10-cm dishes for sucrose gradient centrifugation. Then, siControl or siROR1 were introduced into the cells on the next day, and the cells harvested 3 days after siRNA transfection.

### Ligand and/or TKI treatment in siRNA-treated cells

NCI-H1975 and PC-9 cells were seeded at 1.0 × 10^5^ cells per well in six-well plates, incubated for 1 day, transfected with 20 nM siROR1 or siControl, and incubated for 2 days. The cells were subsequently serum-starved for 24 h, followed by stimulation with 50 ng ml^−1^ of IGF-I, IGF-II, insulin, PDGF or HGF for 30 min. The cells were then harvested for WB analysis (Fig. 1b and Supplementary Fig. 1b).

A total of 1 × 10^5^ cells per well were seeded into six-well plates, transfected with 20 nM siROR1, sicavin-1, siCAV1 or siControl, and then cultured for 3 days before concurrent treatment with the indicated amounts of a TKI and bypass signal-mediating growth factors. We added 1 μM gefitinib, an EGFR–TKI, or 0.5 μM CL-387, 785, an irreversible EGFR–TKI that is effective even in cancer cells carrying gefitinib-resistant T790M double EGFR mutations^28^, together with 50 ng ml^−1^ of IGF-I and HGF, to the culture media of A431, PC-9 and NCI-H1975 cells. The cells were incubated for 6 h and then harvested for WB analysis. To measure the effects on cell proliferation, 5.0 × 10^4^ cells per well in six-well plates were similarly treated and incubated for 4 days before being harvested for a colorimetric assay. NCI-H1975 cells were also treated with SRC kinase inhibitors (dasatinib; 1 μM, SKI-I; 5 μM) for 6 h and harvested for WB analysis.

### CAV1 introduction following siROR1 treatment in COS-7 cells

COS-7 cells were seeded at 1.0 × 10^5^ (for WB) or 5.0 × 10^4^ (for immunofluorescence) cells per well in six-well plates, incubated for 1 day, and transfected with 20 nM siROR1 or siControl. The cells were incubated for 2 days and then transfected with VC (pCMVpuro-VC-myc) or CAV1 (pCMVpuro-CAV1-myc). The cells were cultured for 24 h and then harvested for WB analysis or immunofluorescence.

### Immunofluorescence microscopy

A total of 5.0 × 10^4^ of NCI-H1975 cells were plated onto coverslips in six-well plates 1 day before siRNA transfection. The cells were fixed 3 days after transfection with PBS containing 3.7% paraformaldehyde at room temperature. The fixed cells were permeabilized with PBS containing 0.1% Triton X-100. Non-specific binding was blocked by incubating the coverslips for 30 min in PBS containing 1.0% BSA (Roche). The fixed cells were incubated with primary antibodies (anti-CAV1, anti-CAV2, anti-ROR1, anti-cavin-1, anti-LAMP-1, or anti-IGF-IR antibodies) diluted in PBS containing 1.0% BSA for 1 h, washed with PBS three times and then incubated with the appropriate secondary antibodies conjugated to the specified Alexa dyes (Invitrogen) for 1 h before mounting with Fluoromount (Diagnostic BioSystems). For dual labelling with anti-cavin-1 and anti-CAV2, the rabbit antibodies were first labelled using either Zenon Alexa Fluor 488 (Z-25302) or 568 (Z-25306) antibody labelling kits (Life Technologies) and then incubated with permeabilized cells for 1 h at room temperature. The cells were incubated with DAPI solution (Dojindo Laboratories) and washed before mounting. Fluorescence was performed using AF6500 fluorescence microscopy (Leica) and Zeiss LSM 880 confocal microscopy (Carl Zeiss, Germany). Colocalization was quantified using ImageJ software. Pearson’s correlation and a scatter diagram were determined by using the colocalization plugin of ImageJ.

Images of the basal plasma membrane were obtained using the ‘unroofing’ method^25^. Briefly, the cells were washed three times with PBS and then overlaid with prewet nitrocellulose paper. The upper half of the cells was removed by peeling off the paper, and the remaining membrane was immediately fixed with 3.7% paraformaldehyde in PBS for 10 min and then labelled with the anti-CAV1 and anti-ROR1 antibodies for immunofluorescence microscopy.

### Super-resolution structured illumination microscopy

NCI-H1975 cells were grown on coverslips (High-performance ISO8255 compliant/No. 1.5H, 170±5 μm, 18 × 18 mm (Carl Zeiss)) and fixed with 3.7% paraformaldehyde in PBS and then permeabilized with PBS containing 0.1% Triton X-100. The fixed cells were incubated with primary antibodies (anti-CAV1 (mouse), anti-ROR1 (goat) and anti-cavin-1 (rabbit) antibodies) diluted in PBS containing 1.0% BSA for 1 h, washed three times with PBS and then incubated with the appropriate secondary antibodies conjugated to the specified Alexa dyes (Alexa-488 (A11055), Alexa-568 (A11031), Alexa-350 (A11046)) for 1 h before mounting with Vectashield H-1,000 (Vector Laboratories, CA). Images were acquired with a Zeiss LSM 880 confocal microscope using an α Plan-Apochromat × 100/numerical aperture 1.46 objective. SIM images were collected on samples obtained with the Zeiss ELYRA PS.1 system (Carl Zeiss Microscopy) using a × 100 objective lens with a numerical aperture of 1.46 at room temperature. Three orientation angles and five phases of the excitation grid were acquired for each Z plane, with Z spacing of 167.2 nm between planes. SIM processing was performed with SIM module of the ZEN 2 software (Carl Zeiss Microscopy).

### Electron microscopy

Cells cultured on gold foils of 20 μm thickness were frozen using an HPM 010 high-pressure freezing machine (Leica). Freeze-fracture replicas prepared using a BAF400 apparatus (Baltec) were treated with SDS, labelled with mouse anti-CAV2 antibody (BD Bioscience) followed by colloidal gold (10 nm)-conjugated goat anti-mouse IgG antibody (British Biocell International) and observed under an JEM-1011 electron microscope (JEOL)^22^,^49^. The distribution density of the caveolar indentation was quantified using randomly taken electron micrographs. The areas of the plasma membranes in the respective micrographs were measured using ImageJ.

The cells cultured and unroofed as previously described were rapidly frozen by plunging them onto a copper block cooled with liquid nitrogen^23^. The frozen samples were placed in the freeze-etching device (FR 9000, Hitachi), and the excess ice covering the samples was removed with pre-chilled glass knives before etching (slight freeze-drying). Etched surfaces were shadowed with platinum and carbon at −93 °C under vacuum at 5 × 10^−6^ Pa.

### Preparation of recombinant proteins

GST-tagged ROR1 (intracellular domain) was expressed in Sf9 insect cells using a Gateway system (Invitrogen) according to the manufacturer’s instructions. Recombinant GST-tagged ROR1-WT protein was purified by glutathione-affinity chromatography. GST, GST-tagged cavin-1-WT, GST-tagged cavin-1-MAD (233–321) and GST-tagged cavin-2-WT were purchased from Abnova (Taipei). Recombinant protein of the GST-tagged CAV1 was also obtained from Abcam (Cambridge). Myc-tagged CAV1 and Myc-tagged cavin-1 were purified from 293T cells transfected with pCMVpuro-cavin-1-WT-myc or pCMVpuro-CAV1-WT-myc using a c-Myc-tagged protein mild purification kit (MBL). Purification was performed according to the manufacturer’s instructions, and the purified proteins were stored at −80 °C for GST pull-down assay.

### GST pull-down assay

Myc-tagged cavin-1 or Myc-tagged CAV1 proteins purified from 293T transfectants were mixed with glutathione beads coated with recombinant GST or GST-tagged ROR1 proteins. After repeated washes with a solution containing 20 mM MOPS (pH7.2), 1 mM dithiothreitol (DTT), 5 mM EGTA, 25 mM β-glycerophosphate, 1 mM Na_3_VO_4_ and 75 mM MgCl_2_, the bound proteins were extracted with SDS sample buffer and subjected to SDS-PAGE, followed by WB analysis with anti-GST or anti-c-Myc antibodies. The NCI-H1975 cells were solubilized in octylglucoside buffer (60 mM octylglucoside, 150 mM NaCl and 50 mM EDTA). The cell extracts were mixed with glutathione beads coated with recombinant GST-tagged ROR1, GST-tagged CAV1, GST-tagged cavin-1-WT, GST-tagged cavin-1-MAD (233–321) or GST-tagged cavin-2. After several rounds of washing, the bound proteins were eluted and subjected to SDS–PAGE followed by WB analysis using anti-GST, anti-ROR1, anti-CAV1 or anti-cavin-1 antibodies.

### Quantitative RT-PCR

Quantitative RT-PCR analysis was carried out by using primers for ROR1, CAV2 and 18S, along with Power SYBR Green PCR Master Mix (Applied Biosystems, Foster City, CA) and an ABI Prism7500 (Applied Biosystems).

### Reproducibility of experiments

The WB scans and electron microscopy and immunofluorescence images shown in this study are representative of the following number of independent experiments with similar results. Figures 1a (left panel), 1c (left panel), 3a–e, 4b, 5b (left panel) 5c–e, 7a, 7c and 7d show representative data from two independent experiments with similar results. Figures 1a (right panel) and 1c (right panel) show data from two independent experiments. Figures 1b, 2b,c,f and 5b (right panel), 6a, 6b, and 8a–c (upper panel) show representative data from three independent experiments with similar results. Figures 2e and 8 (lower panel) show data from three independent experiments. Figures 2a, 4a and 5a (right panel) and 7b show representative data from four independent experiments with similar results. Figure 5a (left panel) show representative data from five independent experiments with similar results. Supplementary Fig. 1a (left panel), 1c (left panel), 2a–c, 3b–d, 5b, 6a, 6b, 7a–c, 8b–d, 8g and 9b show representative data from two independent experiments with similar results. Supplementary Fig. 1a (right panel) and Fig. 1c (right panel) show data from two independent experiments. Supplementary Figs 1b, 2d, 3a, 4a, 5a,c and 8a,e,f show representative data from three independent experiments with similar results. Supplementary Figs 2e and 9a show data from three independent experiments. Supplementary Fig. 4b shows representative data from one experiment.

## Additional information

**How to cite this article:** Yamaguchi, T. *et al.* ROR1 sustains caveolae and survival signalling as a scaffold of cavin-1 and caveolin-1. *Nat. Commun.* 7:10060 doi: 10.1038/ncomms10060 (2016).

## Supplementary Material

Supplementary Information Supplementary Figures 1-11 and Supplementary Tables 1-3
