# Supplementary material for: ROR1 sustains caveolae and survival signalling as a scaffold of cavin-1 and caveolin-1
Source: Nat Commun. 2016 Jan 4;7:10060. doi: 10.1038/ncomms10060 (PMC4777216; doi:10.1038/ncomms10060)
Supplement: Supplementary Information — Supplementary Figures 1-11 and Supplementary Tables 1-3 [file ncomms10060-s1.pdf]

Supplementary Information

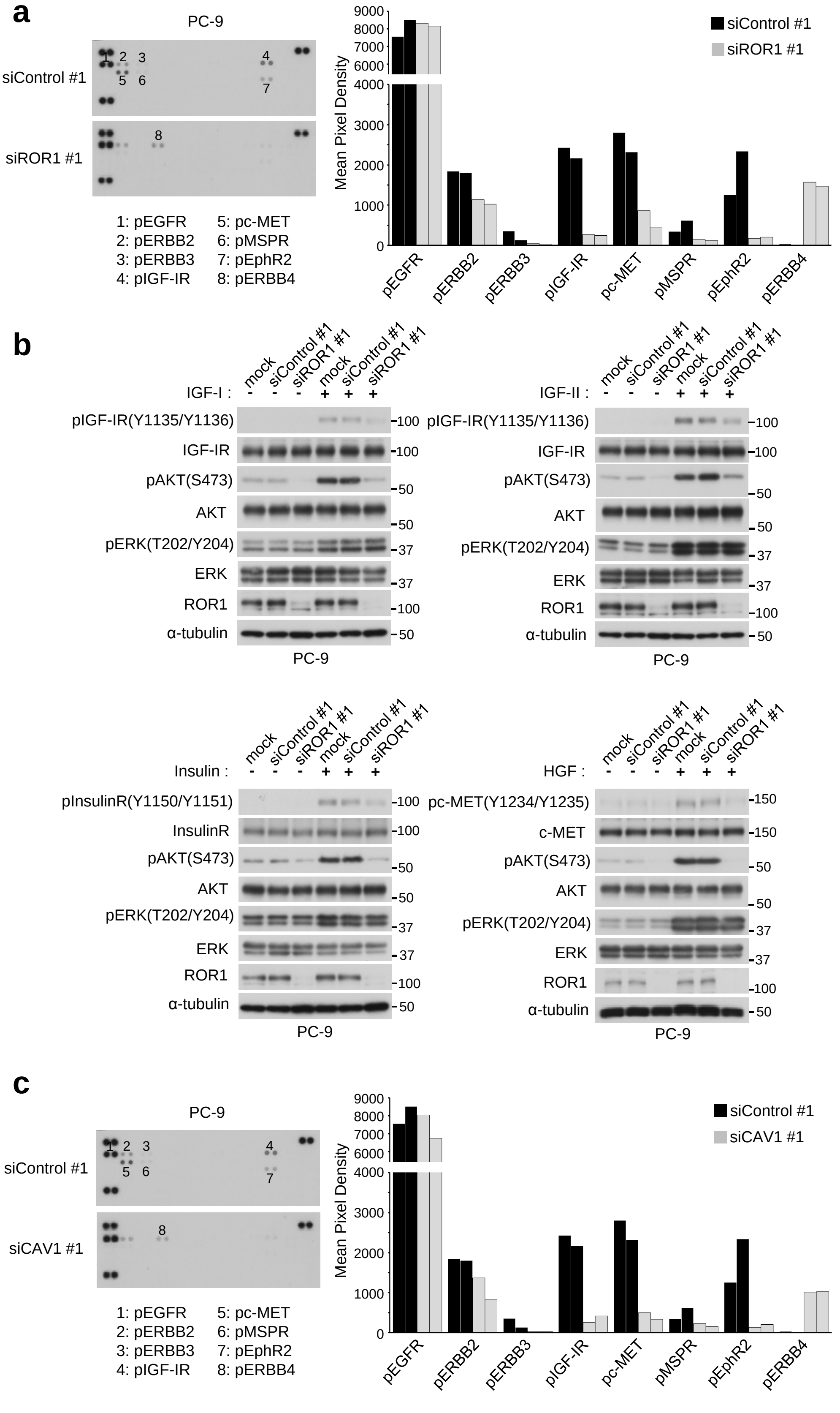

Supplementary Figure 1 | ROR1 and CAV1 knockdown result in decreased phosphorylation of multiple RTKs. (**a**) Phospho-RTK array results showing the inhibitory effects of siROR1 treatment on the phosphorylation state of multiple RTKs in the PC-9 NSCLC cell line (left panel). Averages of the mean pixel densities obtained in two independent experiments are given as two bars for each of the representative RTKs (right panel). (**b**) Impairment of the growth factor-induced phosphorylation of multiple RTKs in PC-9 cells knocked down for ROR1. (**c**) Phospho-RTK array results showing the inhibitory effects of siCAV1 treatment on the phosphorylation state of multiple RTKs in PC-9 cells (left panel) and averages of the mean pixel densities of the representative RTKs in two independent experiments (right panel). The siControl blot of Supplementary Figure 1a is included for ease of comparison. Uncropped images of blots are shown in Supplementary Figure 11.

Supplementary Information

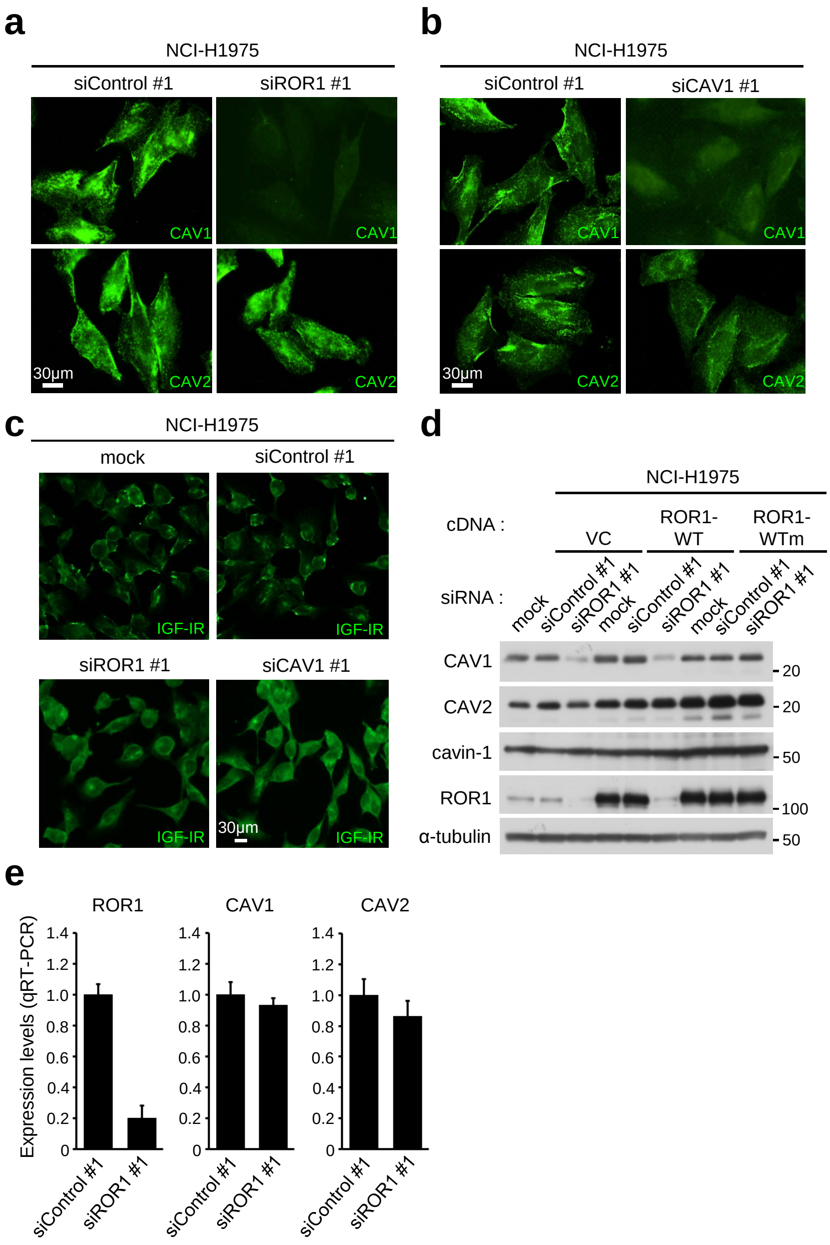

Supplementary Figure 2 | ROR1 knockdown inhibits the expression of CAV1 protein but not of CAV1 mRNA and results in caveolae loss. (**a**) Decreased CAV1 but not CAV2 expression, observed by immunofluorescence staining in siROR1-treated NCI-H1975. (**b**) The retention of CAV2 expression, observed by immunofluorescence staining in siCAV1-treated NCI-H1975 cells. (**c**) Immunofluorescence analysis showing the altered distributions of IGF-IR in siROR1- and siCAV1-treated NCI-H1975 cells. (**d**) WB analysis verifying the specificity of siROR1 in relation to its effect on CAV1 expression. (**e**) Quantitative RT-PCR assay results showing no effects of siROR1 treatment on CAV1 and CAV2 mRNA expression. Uncropped images of blots are shown in Supplementary Figure 11.

Supplementary Information

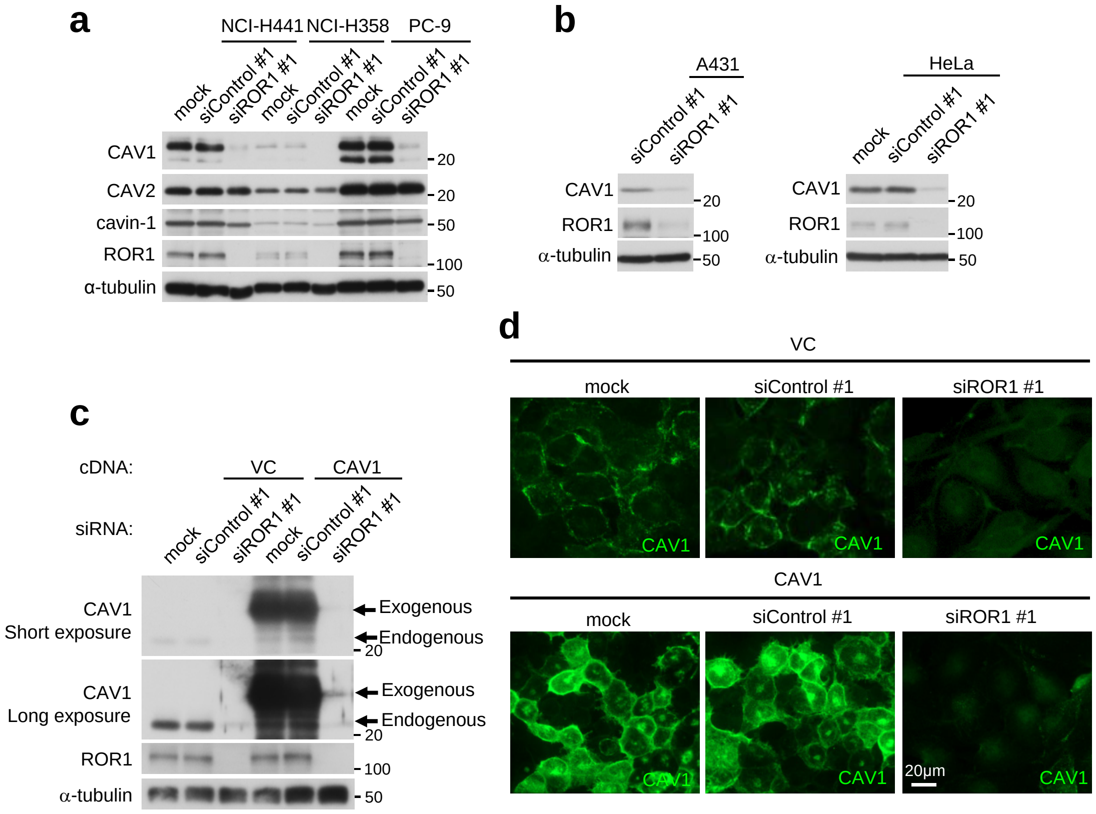

Supplementary Figure 3 | ROR1 is required for CAV1 expression. (**a**) Decreased expression of CAV1 but not CAV2 shown in WB analysis of the NCI-H441, NCI-H358 and PC-9 NSCLC cell lines treated with siROR1. (**b**) WB analysis showing decreased CAV1 expression in the A431 vulval epidermoid carcinoma cell line as well as in the HeLa cervical cancer cell line. (**c**) WB showing decreased expression of both endogenous and exogenous CAV1 in COS-7 cells knocked down for ROR1. (**d**) Decreased CAV1 expression, shown by immunofluorescence staining of siROR1-treated COS-7 cells. Note that exogenously introduced, myc-tagged CAV1 also requires ROR1, similarly to endogenous CAV1. Uncropped images of blots are shown in Supplementary Figure 11.

Supplementary Information

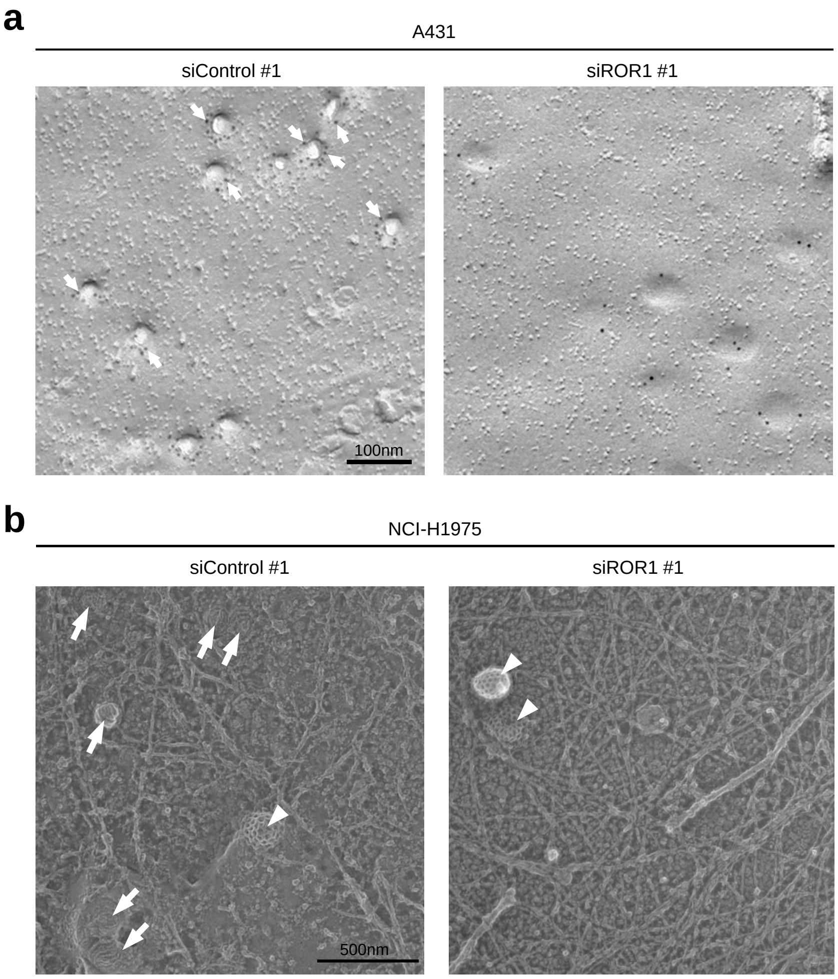

Supplementary Figure 4 | ROR1 knockdown significantly inhibits caveolae formation. (**a**) Representative results of freeze-fracture immunoelectron microscopy in siROR1-treated A431 cells. (**b**) Representative results of freeze-etching electron microscopy showing a lack of typical caveolae structures (in contrast to the retention of clathrin-coated pits) in siROR1-treated NCI-H1975 cells. Note that the cytoplasmic surface of the cell membranes can be observed in this analysis. Arrows, caveolae; arrowheads, clathrin-coated pits.

Supplementary Information

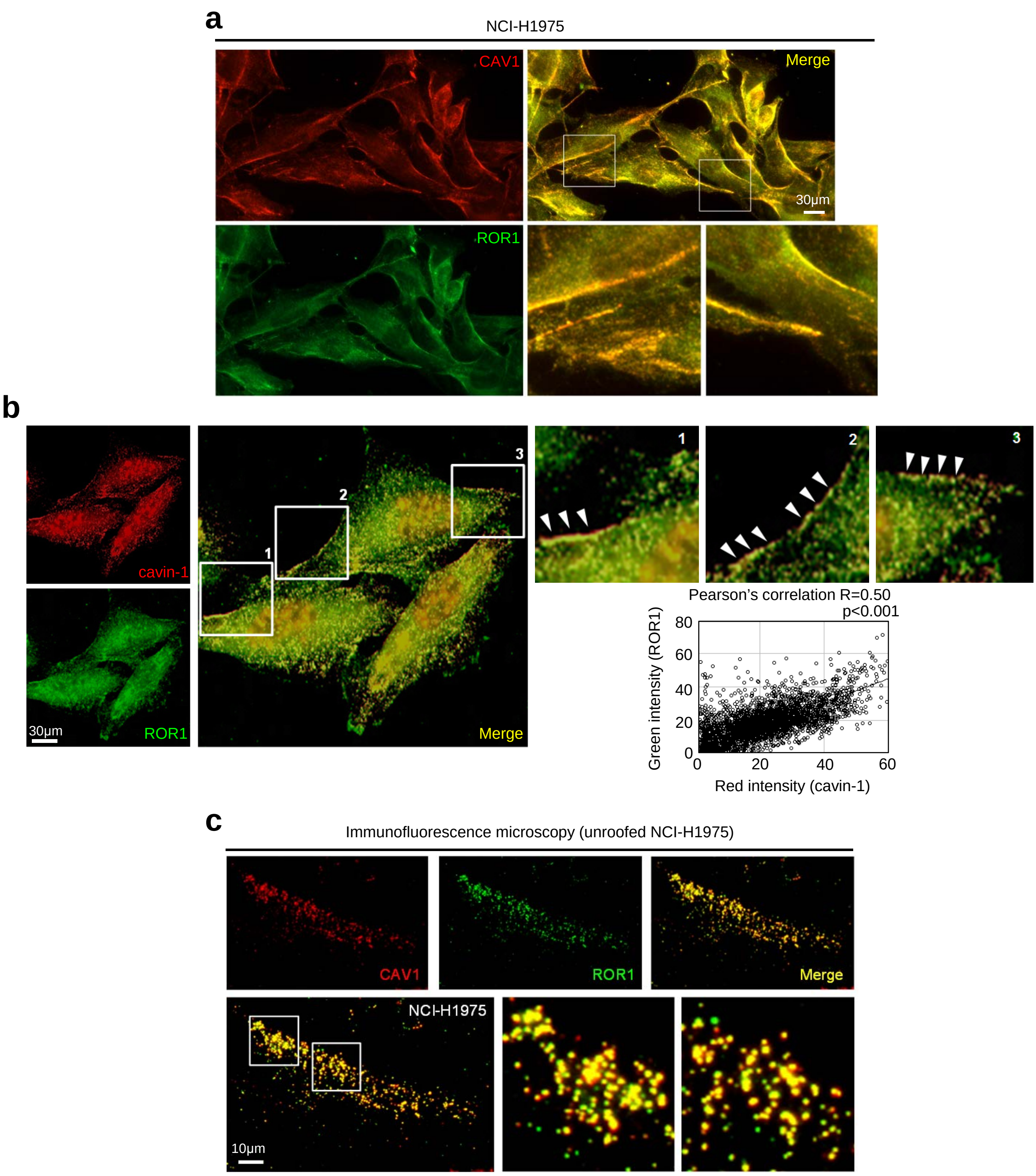

Supplementary Figure 5 | ROR1 is colocalized with CAV1 and cavin-1. (**a**) Two-color immunofluorescence staining results at lower magnification showing the co-localization between ROR1 and CAV1 in NCI-H1975 cells. (**b**) Two-color immunofluorescence staining results showing the co-localization of the punctate signals of ROR1 and cavin-1 in NCI-H1975. The colocalization was quantified using Image J software. (**c**) Two-color immunofluorescence staining combined with the unroofing procedure in NCI-H1975 cells.

Supplementary Information

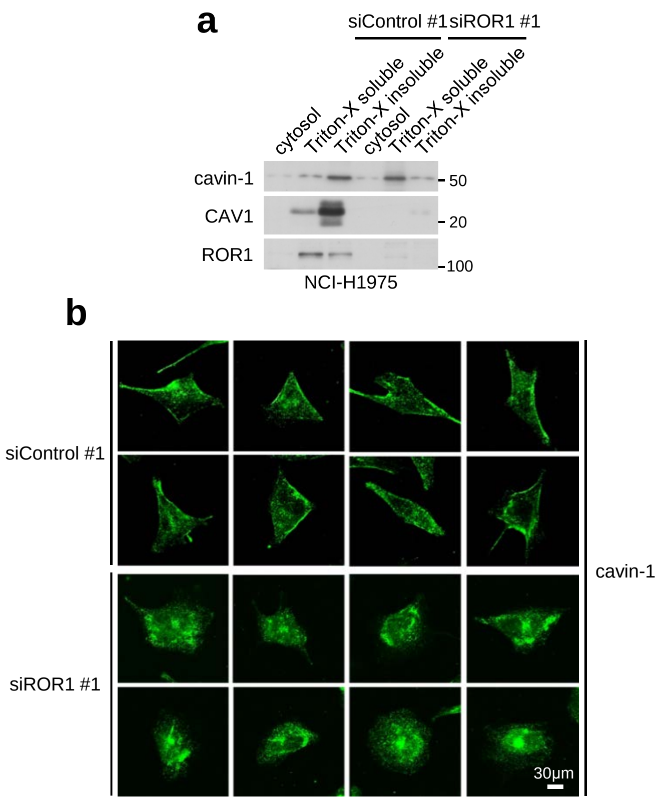

Supplementary Figure 6 | CAV1 expression and cavin-1 subcellular localization are altered by ROR1 knockdown. (**a**) A marked decrease in CAV1 expression and the near-complete loss of cavin-1 from the Triton X-100-insoluble fraction in siROR1-treated NCI-H1975. (**b**) Immunofluorescence staining results showing altered distribution of the punctate signals of cavin-1. Uncropped images of blots are shown in Supplementary Figure 11.

Supplementary Information

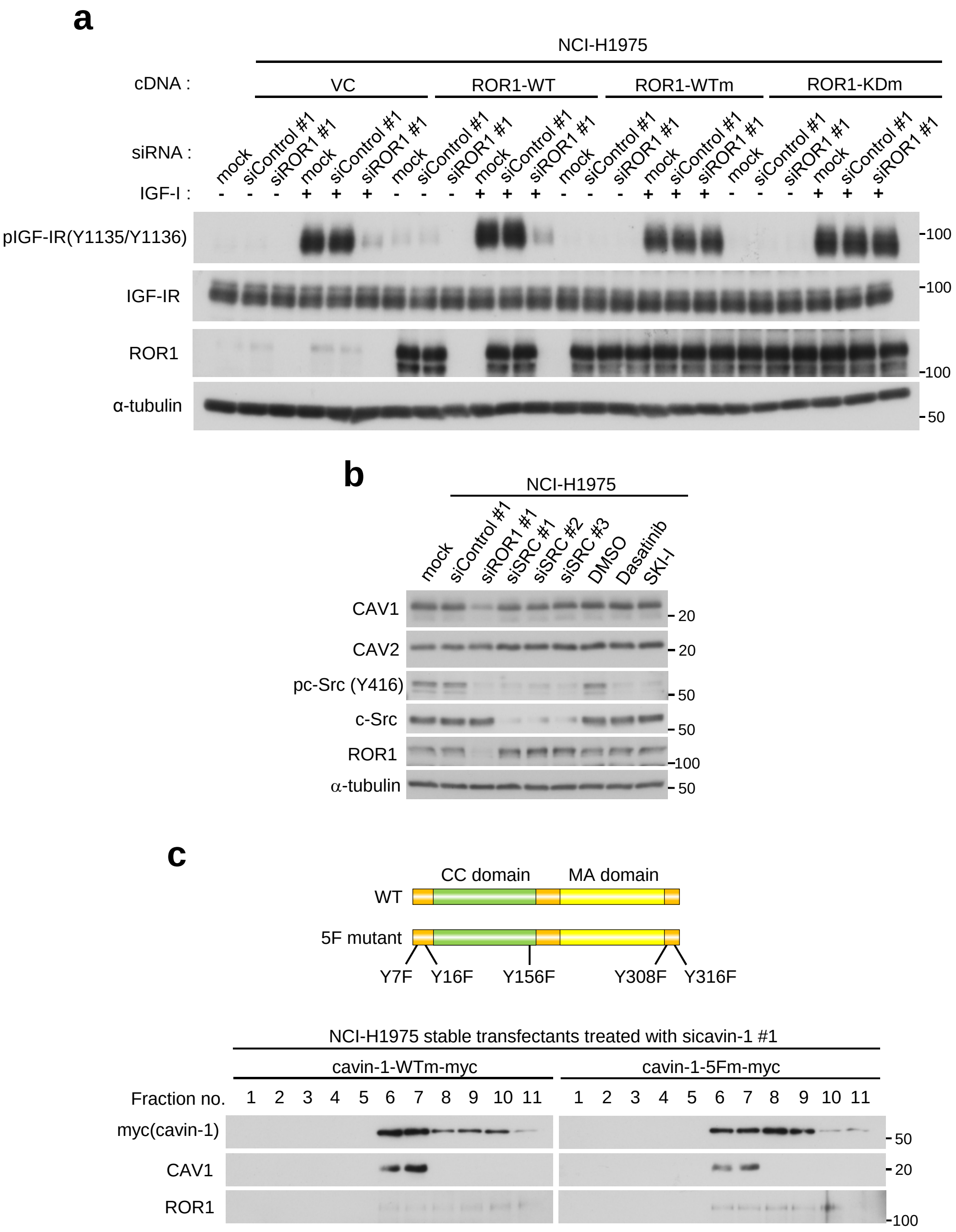

Supplementary Figure 7 | The sustainment of IGF-IR signaling and CAV1 expression does not require ROR1 and SRC kinase activities. (**a**) WB analysis showing the sustainment of IGF-I-stimulated IGF-IR phosphorylation in the presence of kinase-dead ROR1. (**b**) WB analysis showing the sustainment of CAV1 expression in the presence of SRC kinase inhibitors. (**c**) Sucrose gradient centrifugation analysis showing no effects of the replacement of all five potential tyrosine phosphorylation sites of cavin-1 on its subcellular compartmentalization. Uncropped images of blots are shown in Supplementary Figure 11.

Supplementary Information

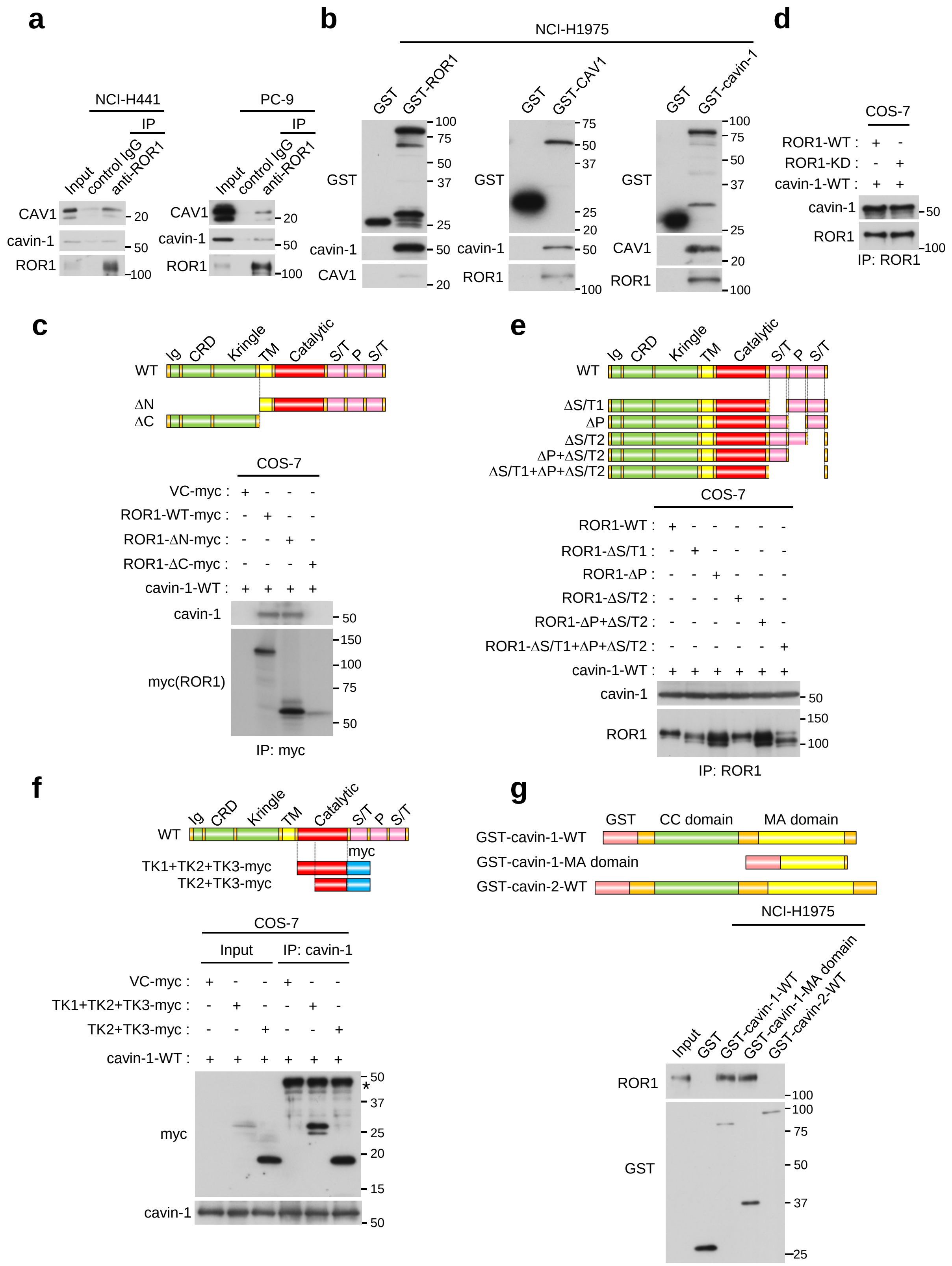

Supplementary Figure 8 | ROR1 interacts with cavin-1 and CAV1. (**a**) IP-WB analysis showing the interaction of endogenous ROR1 with CAV1 and cavin-1 in NCI-H441 and PC-9 cells. (**b**) Mutual interactions among ROR1, cavin-1 and CAV1 detected by pull-down assays using purified GST-tagged proteins and NCI-H1975 cell lysates. (**c**) IP-WB analysis of COS-7 cells introduced with either wt-ROR1 or ROR1 deletion mutants together with cavin-1 showing an interaction of the intracellular domain of ROR1 with cavin-1. (**d**) The interaction of cavin-1 with both wt and kinase-dead ROR1, shown by IP-WB analysis of COS-7 cells co-transfected with cavin-1 and either wt or kinase-dead ROR1. (**e**) The lack of interaction of cavin-1 with any of the two serine/threonine-rich and proline-rich domains shown by IP-WB analysis of COS-7 cells co-transfected with cavin-1 and various ROR1 deletion mutants. (**f**) Interaction of the C-terminal two-thirds of the ROR1 kinase domain with cavin-1, shown by IP-WB analysis of transiently co-transfected COS-7 cells. An asterisk indicates a non-specific signal due to IgG. (**g**) Interaction of the membrane association domain of cavin-1 with ROR1, shown by a pull-down assay using purified GST-tagged cavin-1 proteins and NCI-H1975 cell lysates. Uncropped images of blots are shown in Supplementary Figure 11.

Supplementary Information

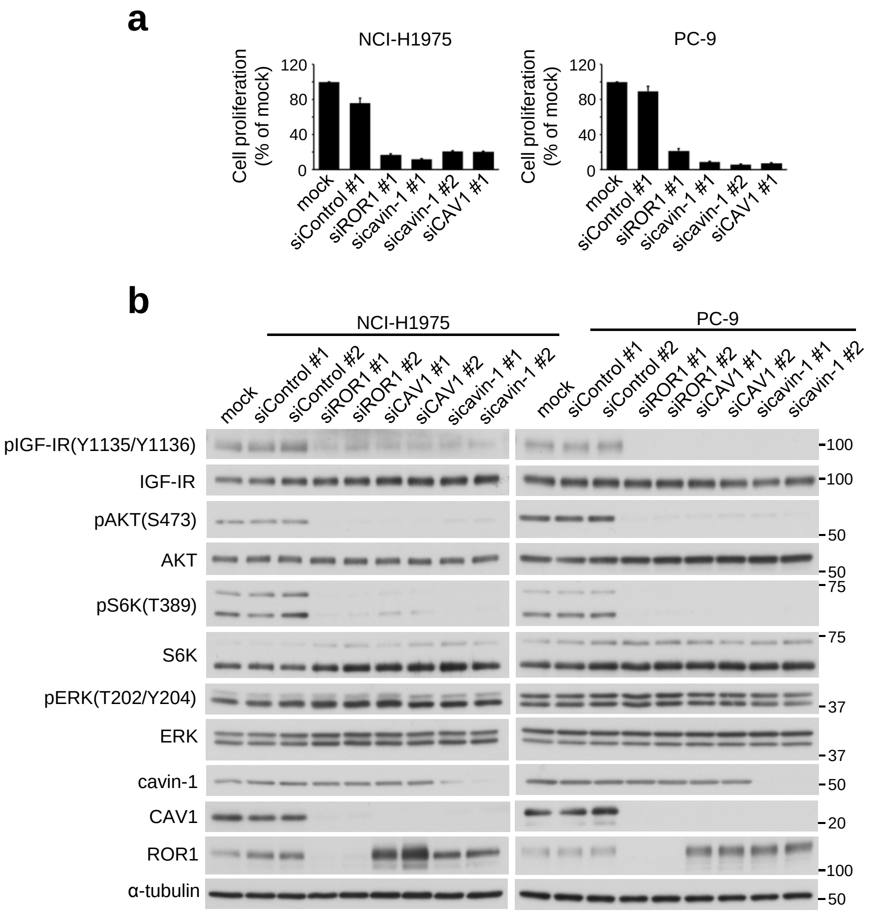

Supplementary Figure 9 | Cell proliferation and IGF-IR, AKT and S6K phosphorylation are inhibited with siROR1 as well as with sicavin-1 and siCAV1. (**a**) MTT assay results showing significant cell proliferation inhibition by siROR1, sicavin-1 or siCAV1 treatment in NCI-H1975 and PC-9 cells. (**b**) Decreased IGF-IR, AKT and S6K phosphorylation, shown by WB analysis of NCI-H1975 and PC-9 cells treated with siROR1, sicavin-1 or siCAV1. Uncropped images of blots are shown in Supplementary Figure 11.

Supplementary Information

a

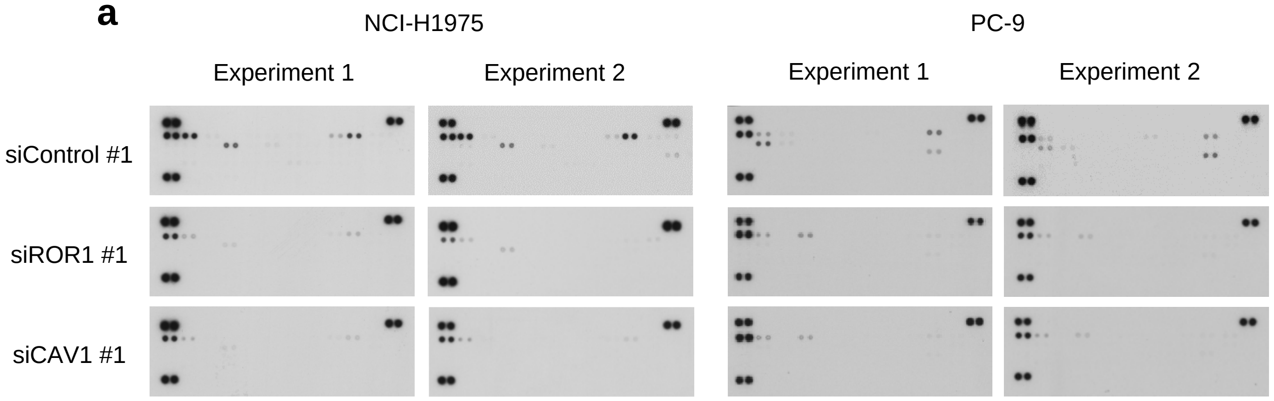

b

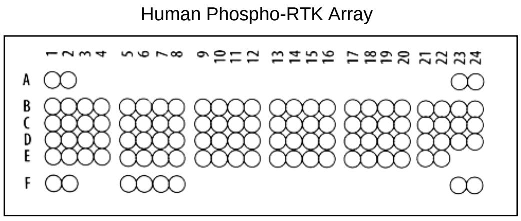

Coordinate	Receptor Family	RTK/Control	Coordinate	Receptor Family	RTK/Control
A1, A2	Reference Spots	-	D1, D2	Tie	Tie-2
A23, A24	Reference Spots	-	D3, D4	NGF R	TrkA
B1, B2	EGF R	EGF R	D5, D6	NGF R	TrkB
B3, B4	EGF R	ErbB2	D7, D8	NGF R	TrkC
B5, B6	EGF R	ErbB3	D9, D10	VEGF R	VEGF R1
B7, B8	EGF R	ErbB4	D11, D12	VEGF R	VEGF R2
B9, B10	FGF R	FGF R1	D13, D14	VEGF R	VEGF R3
B11, B12	FGF R	FGF R2α	D15, D16	MuSK	MuSK
B13, B14	FGF R	FGF R3	D17, D18	Eph R	EphA1
B15, B16	FGF R	FGF R4	D19, D20	Eph R	EphA2
B17, B18	Insulin R	Insulin R	D21, D22	Eph R	EphA3
B19, B20	Insulin R	IGF-I R	D23, D24	Eph R	EphA4
B21, B22	Axl	Axl	E1, E2	Eph R	EphA6
B23, B24	Axl	Dtk	E3, E4	Eph R	EphA7
C1, C2	Axl	Mer	E5, E6	Eph R	EphB1
C3, C4	HGF R	HGF R	E7, E8	Eph R	EphB2
C5, C6	HGF R	MSP R	E9, E10	Eph R	EphB4
C7, C8	PDGF R	PDGF Rα	E11, E12	Eph R	EphB6
C9, C10	PDGF R	PDGF Rβ	E13, E14	Insulin R	ALK
C11, C12	PDGF R	SCF R	E15, E16	-	DDR1
C13, C14	PDGF R	Flt-3	E17, E18	-	DDR2
C15, C16	PDGF R	M-CSF R	E19, E20	Eph R	EphA5
C17, C18	RET	c-Ret	E21, E22	Eph R	EphA10
C19, C20	ROR	ROR1	F1, F2	Reference Spots	-
C21, C22	ROR	ROR2	F5, F6	Eph R	EphB3
C23, C24	Tie	Tie-1	F7, F8	-	RYK
			F23, F24	Control (-)	PBS

Supplementary Figure 10 | Original data from phospho-RTK array analysis in NCI-H1975 and PC-9 cells. (**a**) Scans of phospho-RTK arrays in two independent experiments. (**b**) Spot locations of each anti-phospho-RTK antibody on the array membrane. Uncropped images of blots are shown in Supplementary Figure 11.

Supplementary Information

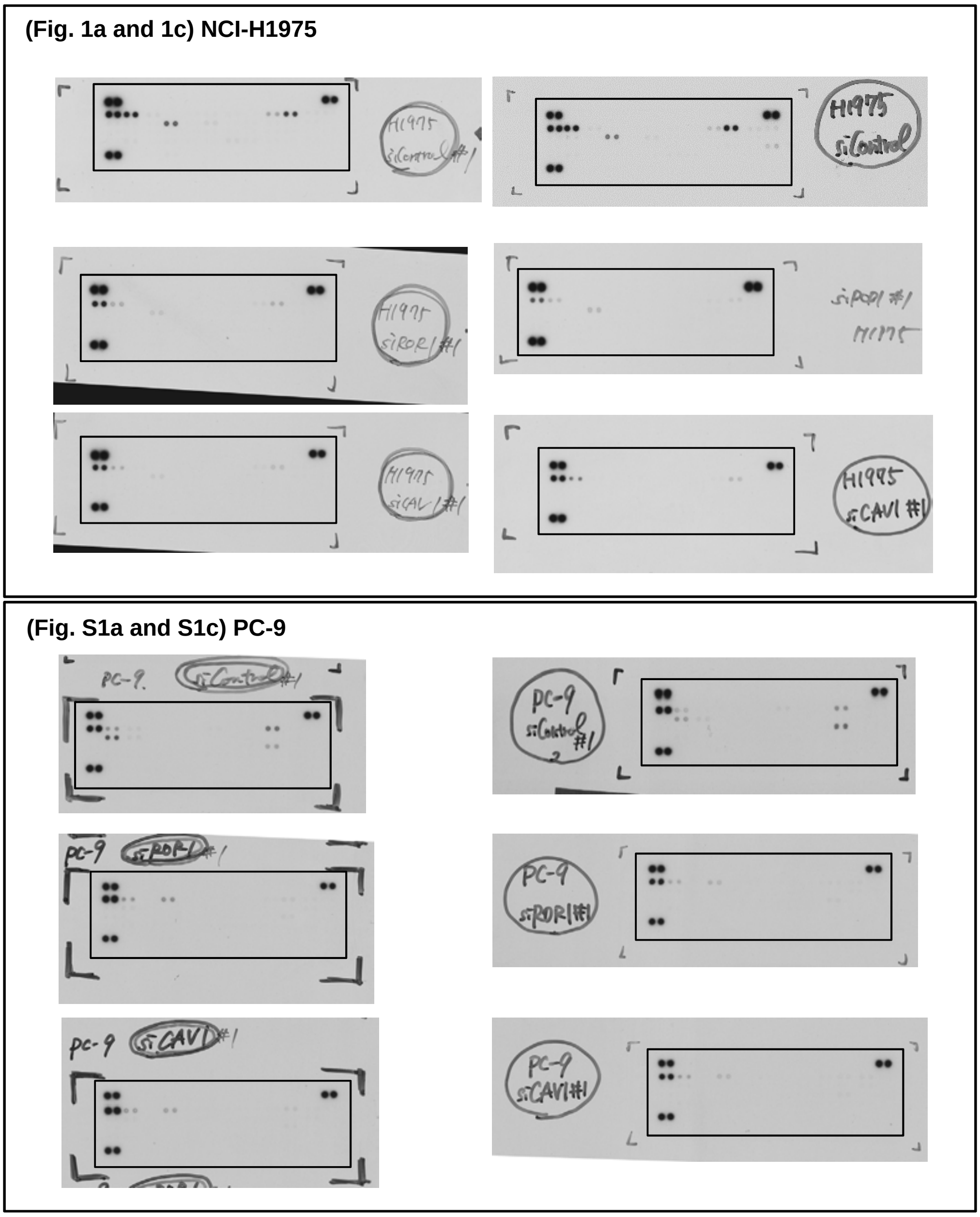

Supplementary Figure 11 | Original uncropped western blots.

Supplementary Information

(Fig. 1b) IGF-I treatment

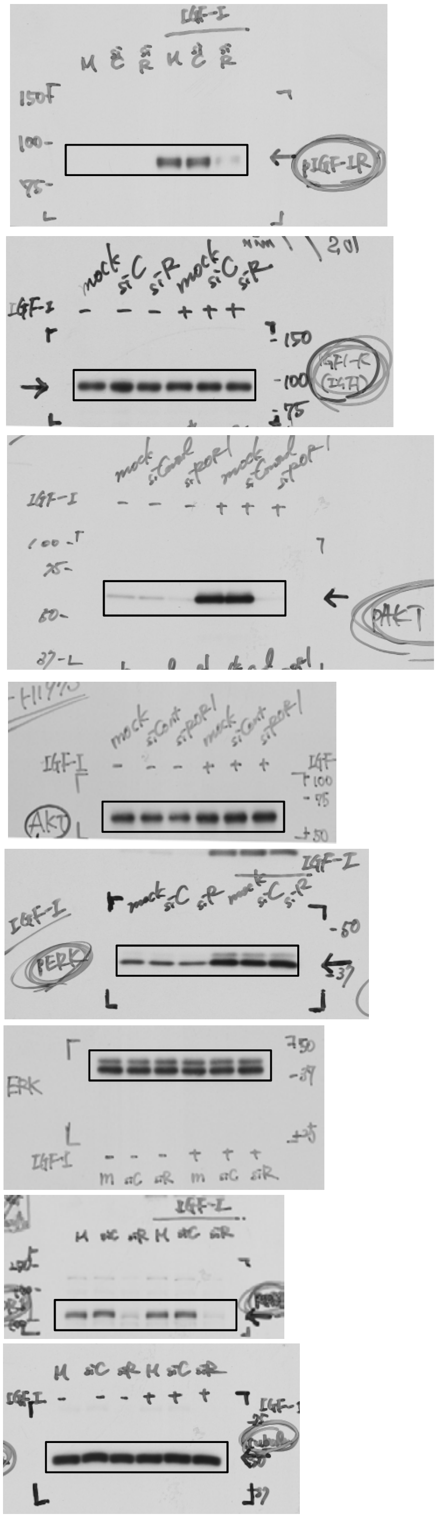

(Fig. 1b) IGF-II treatment

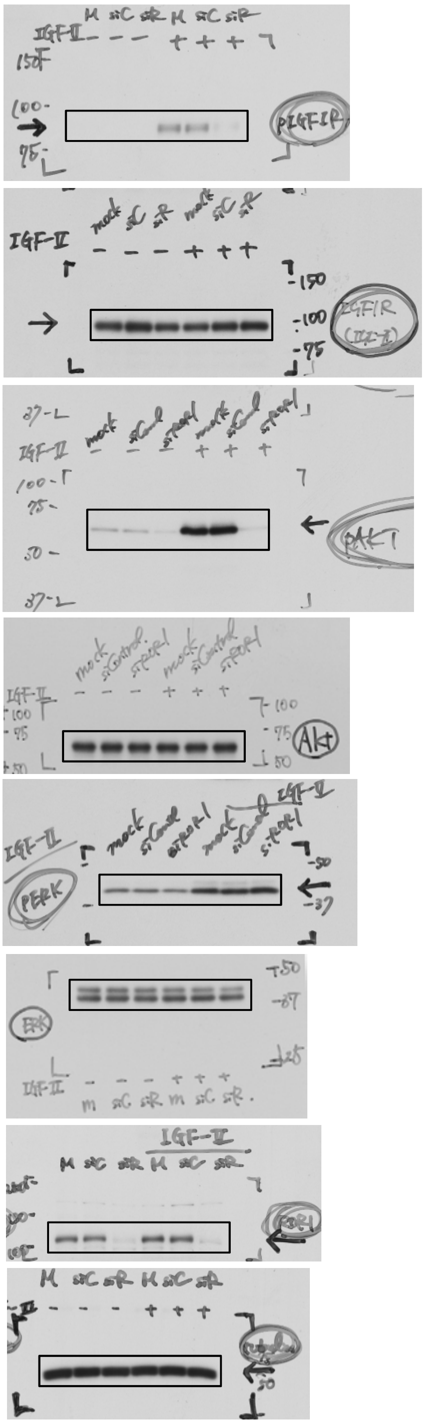

(Fig. 1b) Insulin treatment

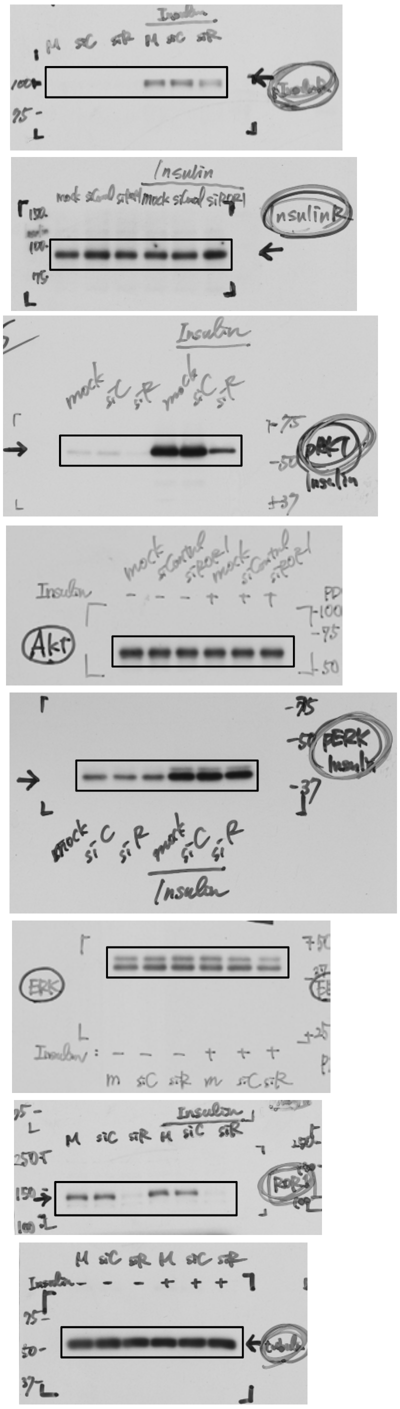

Supplementary Figure 11 | Original uncropped western blots.

Supplementary Information

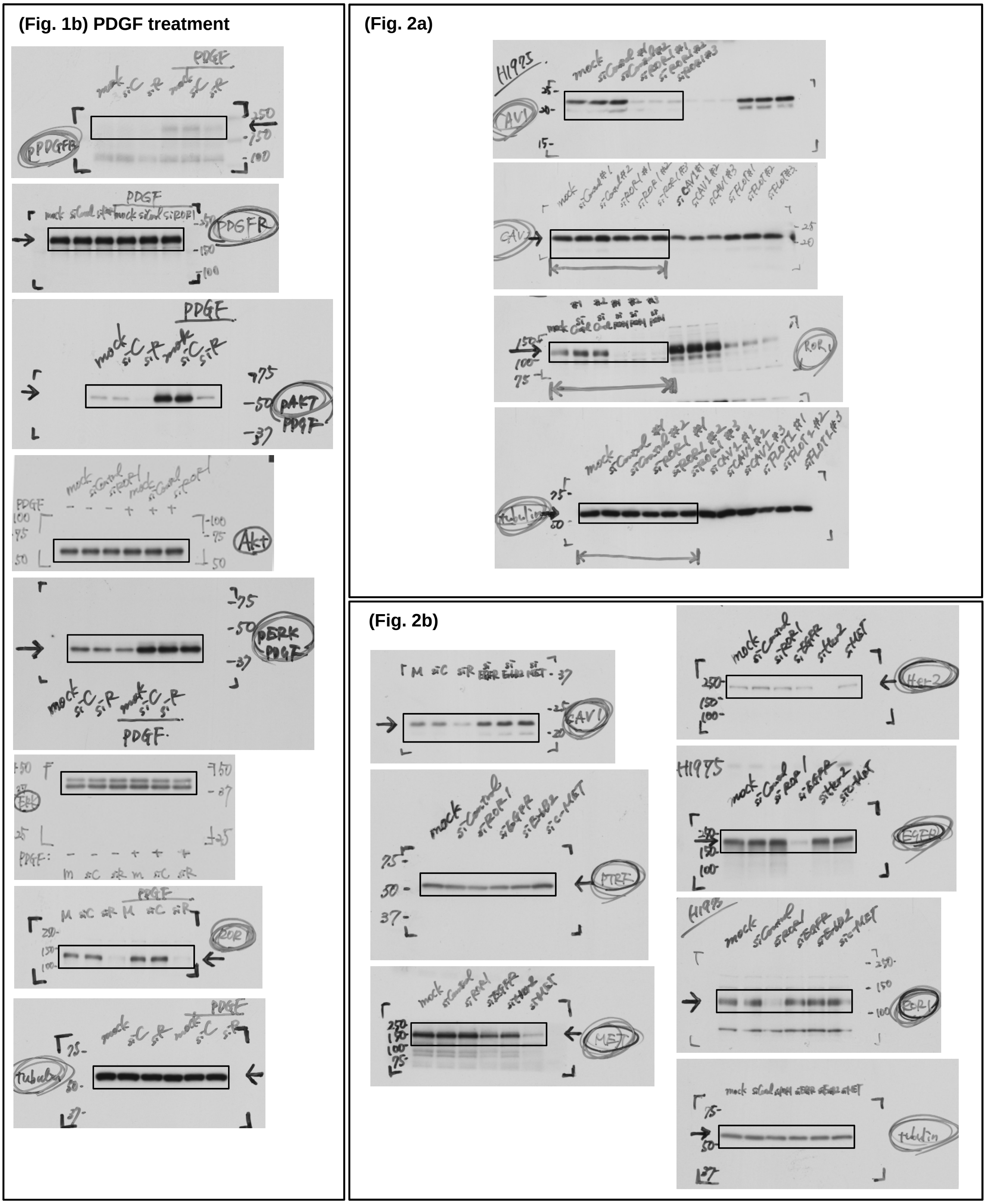

Supplementary Figure 11 | Original uncropped western blots.

Supplementary Information

(Fig. 2c) chloroquine treatment

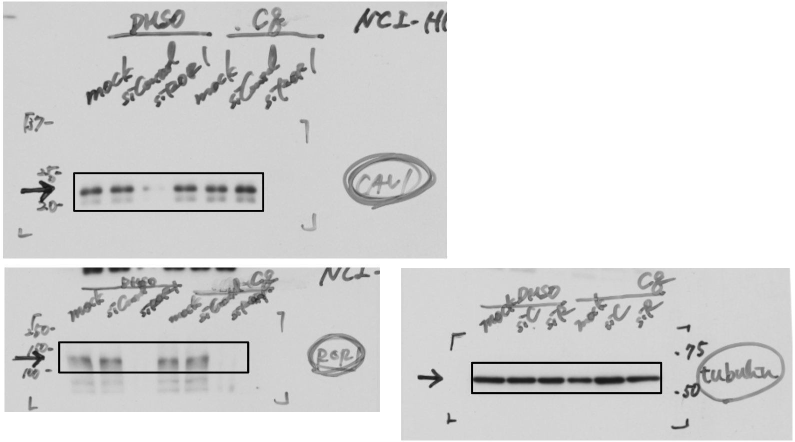

(Fig. 2c) MG262 treatment

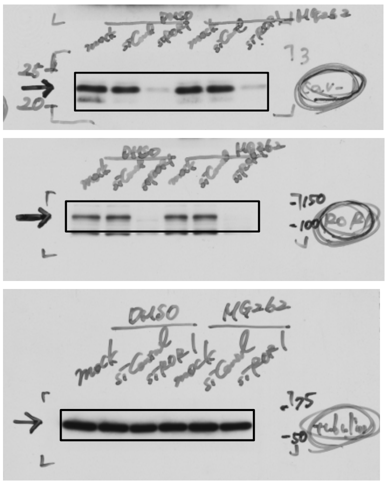

(Fig. 3a)

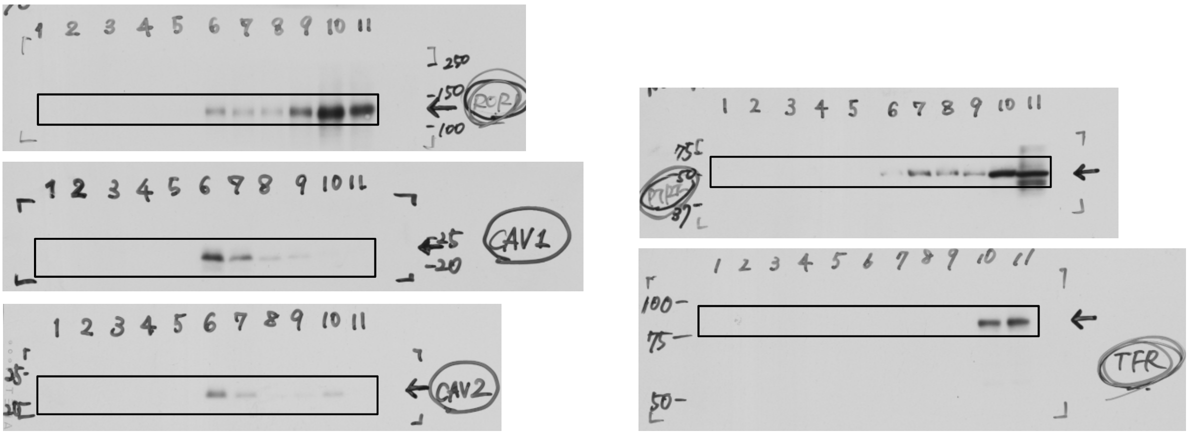

(Fig. 3d) siControl

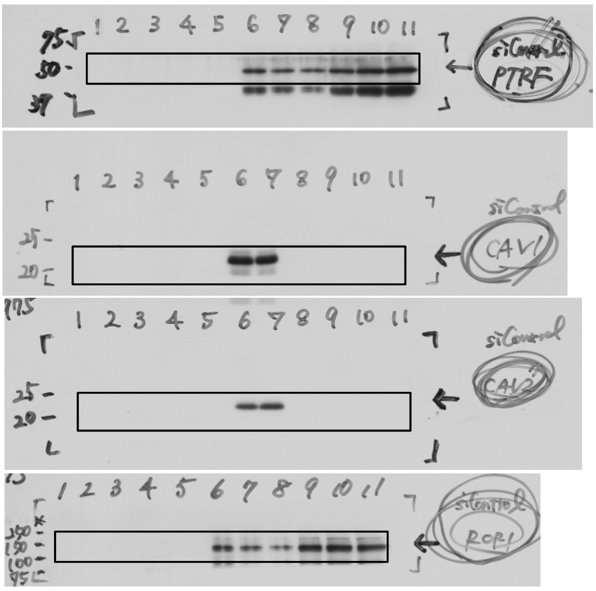

(Fig. 3d) siROR1

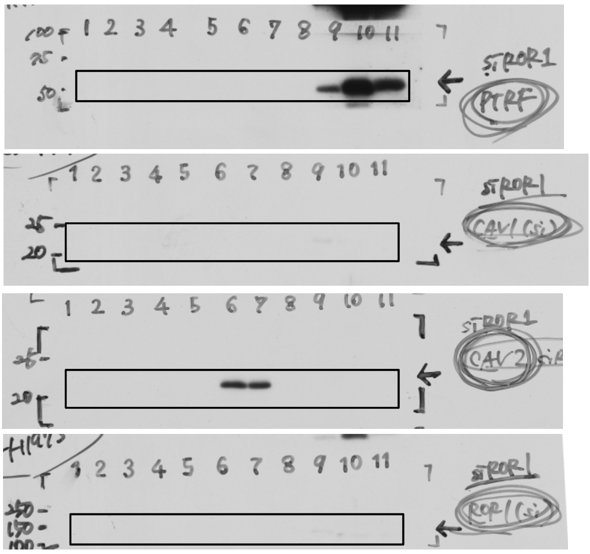

Supplementary Figure 11 | Original uncropped western blots.

Supplementary Information

(Fig. 4a)

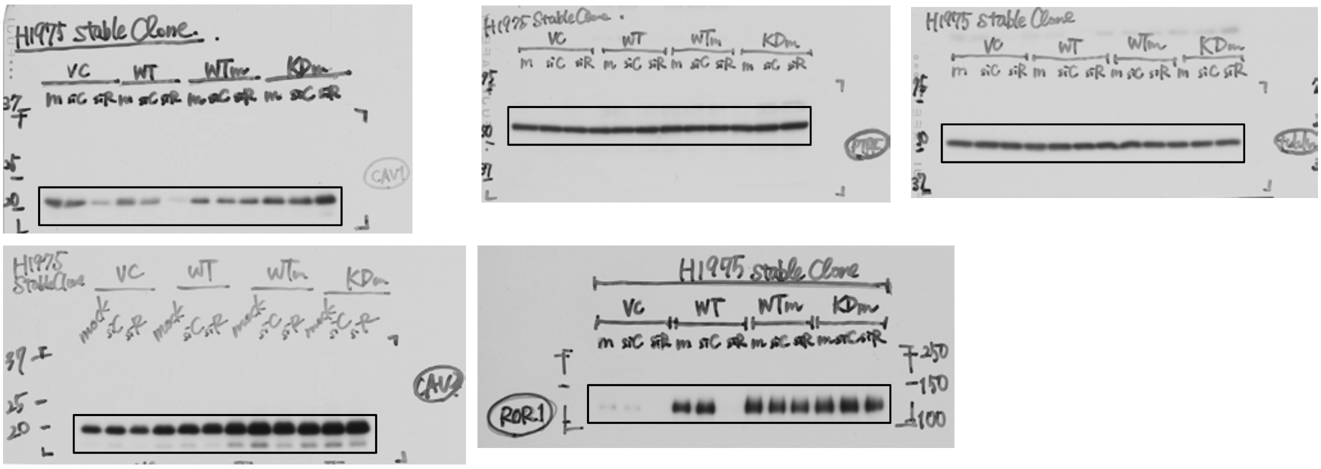

(Fig. 4b) ROR1-WTm

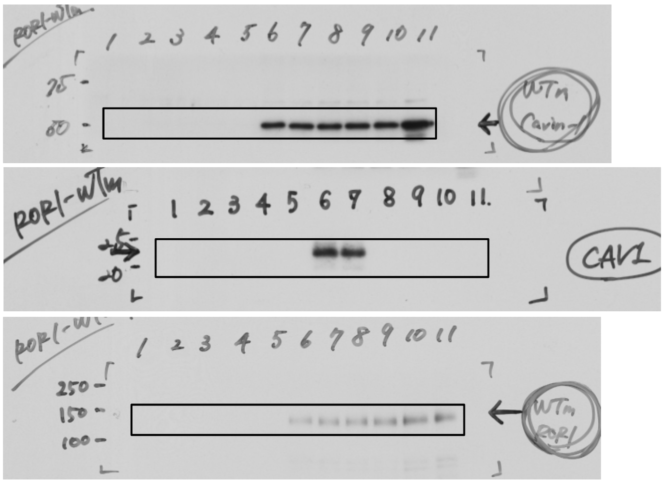

(Fig. 4b) ROR1-KDm

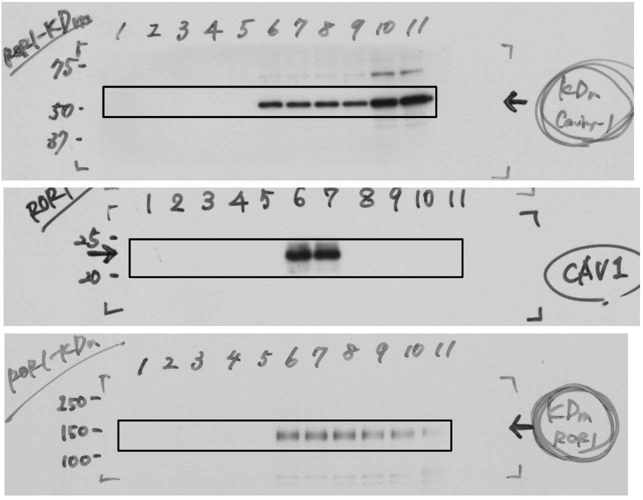

Supplementary Figure 11 | Original uncropped western blots.

Supplementary Information

(Fig. 5a)
NCI-H1975

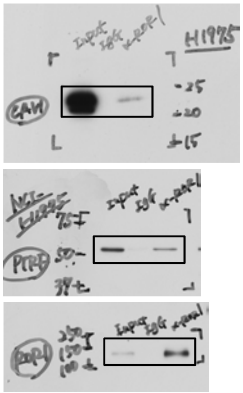

(Fig. 5a)
SK-LU-1

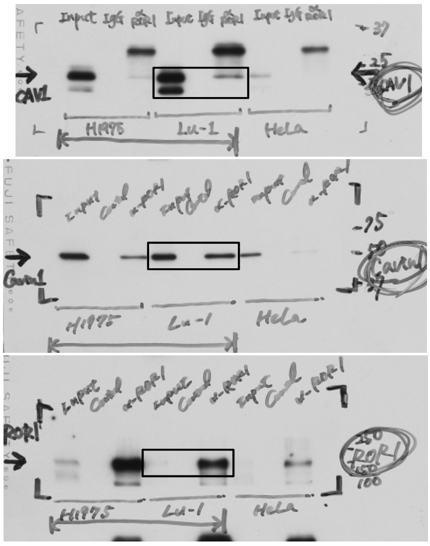

(Fig. 5b)
Left panel

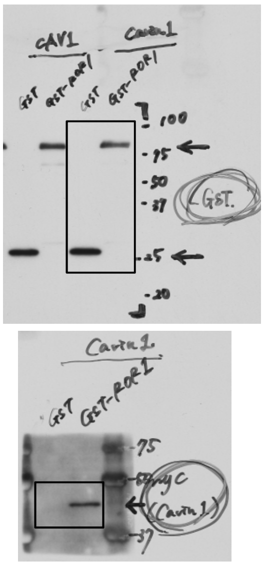

(Fig. 5b)
Right panel

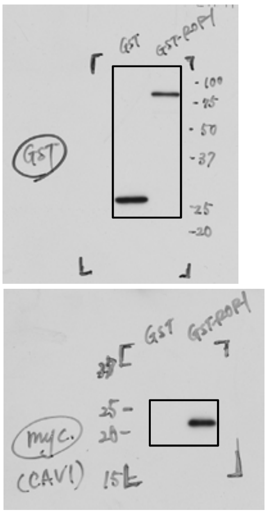

(Fig. 5c)

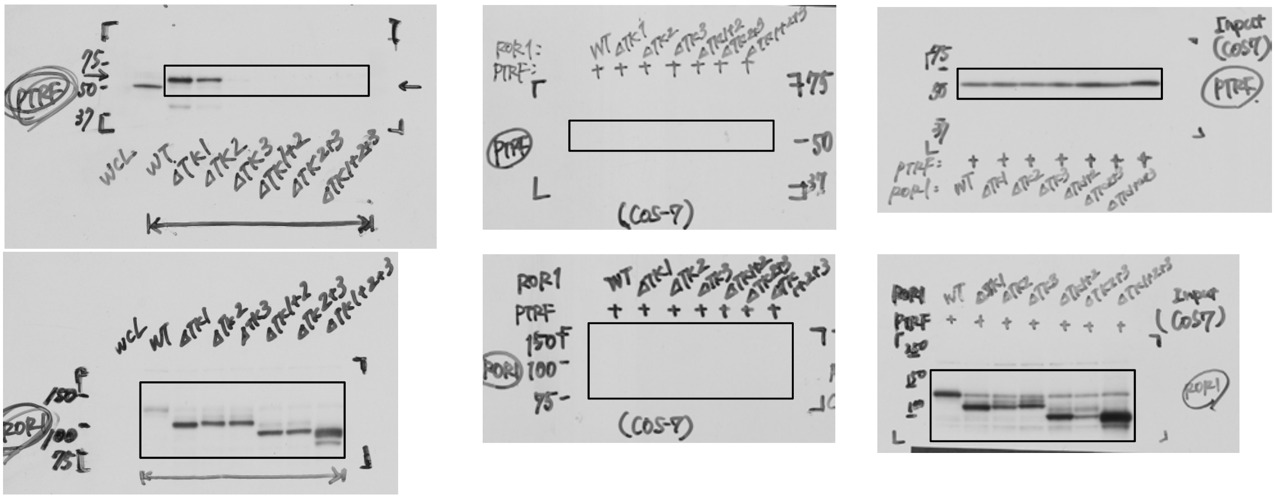

(Fig. 5d)

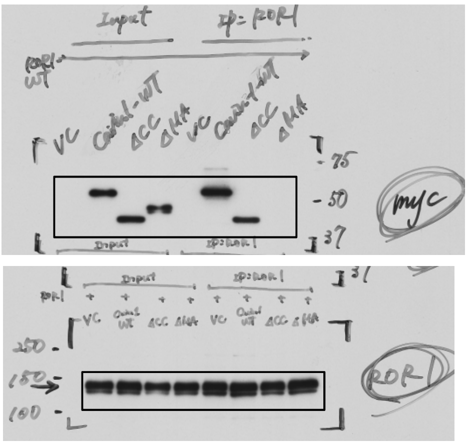

Supplementary Figure 11 | Original uncropped western blots.

Supplementary Information

(Fig. 5e)

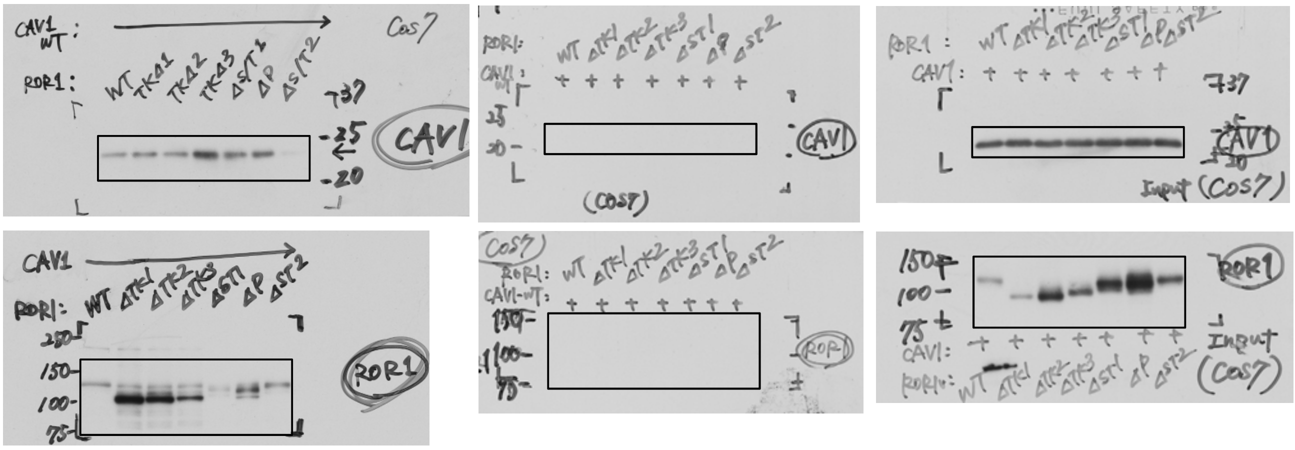

(Fig. 6a)

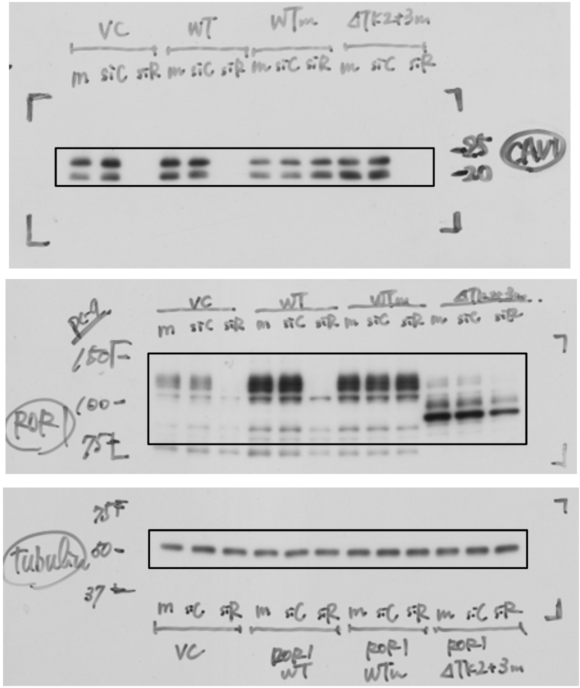

(Fig. 6b)

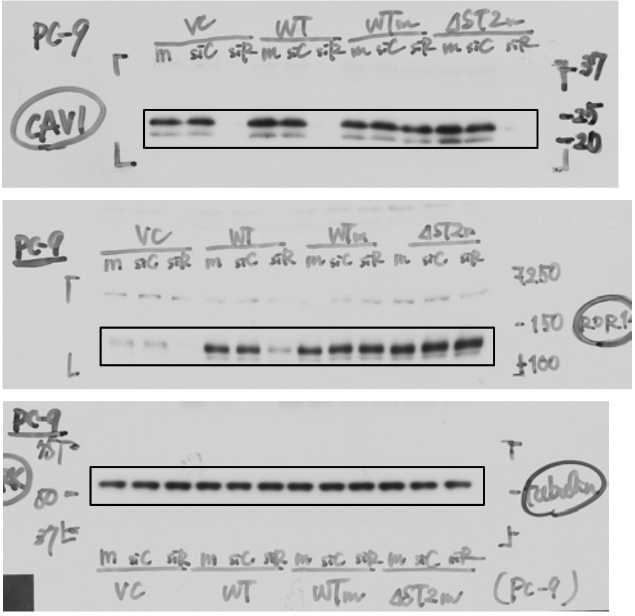

Supplementary Figure 11 | Original uncropped western blots.

Supplementary Information

(Fig. 7a) siControl

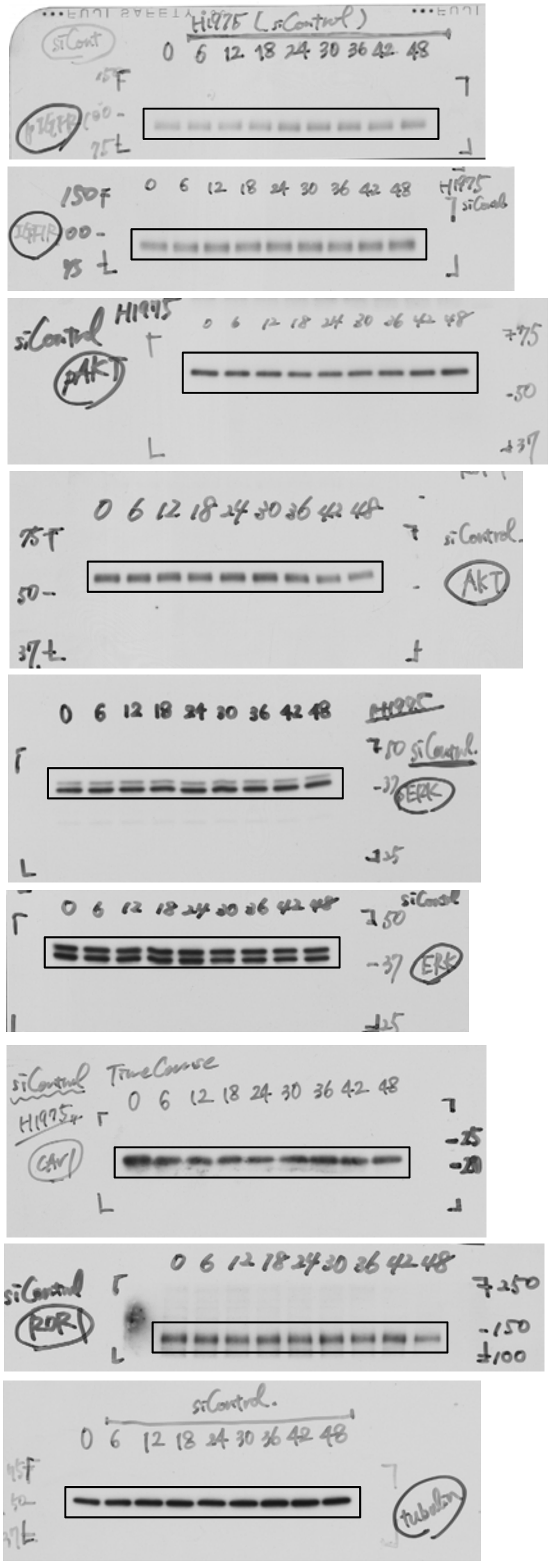

(Fig. 7a) siROR1

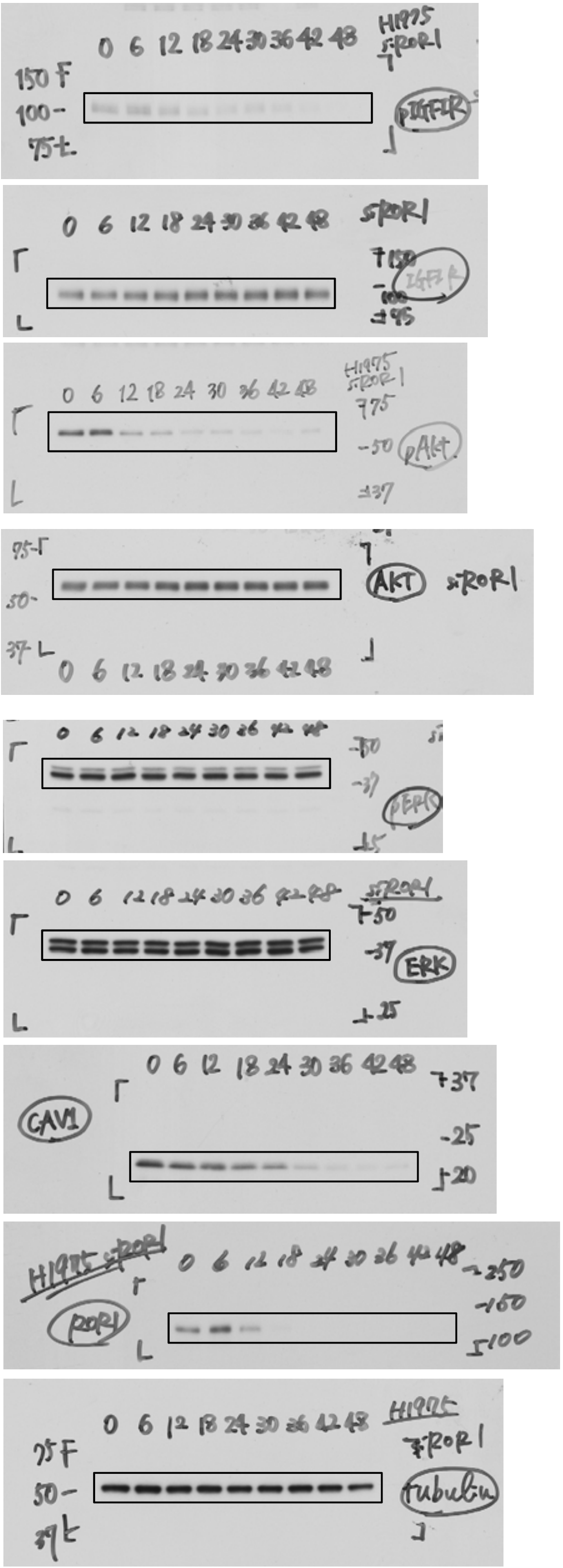

Supplementary Figure 11 | Original uncropped western blots.

Supplementary Information

(Fig. 7b)

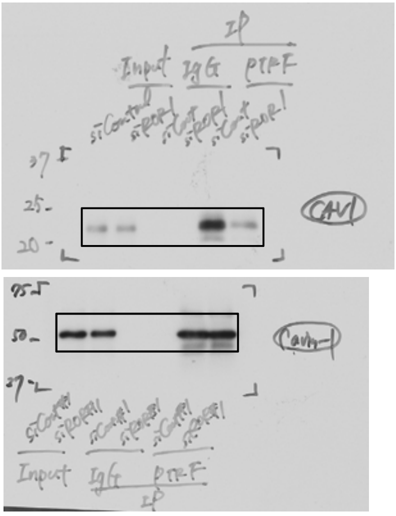

(Fig. 8a)

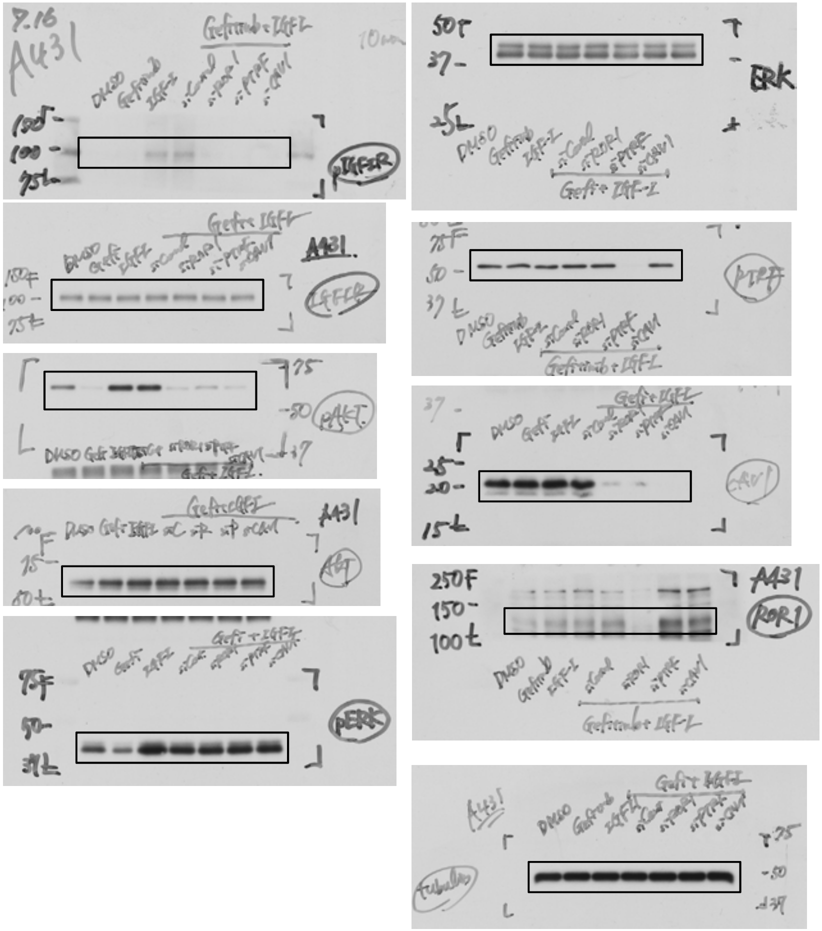

Supplementary Figure 11 | Original uncropped western blots.

Supplementary Information

(Fig. 8b)

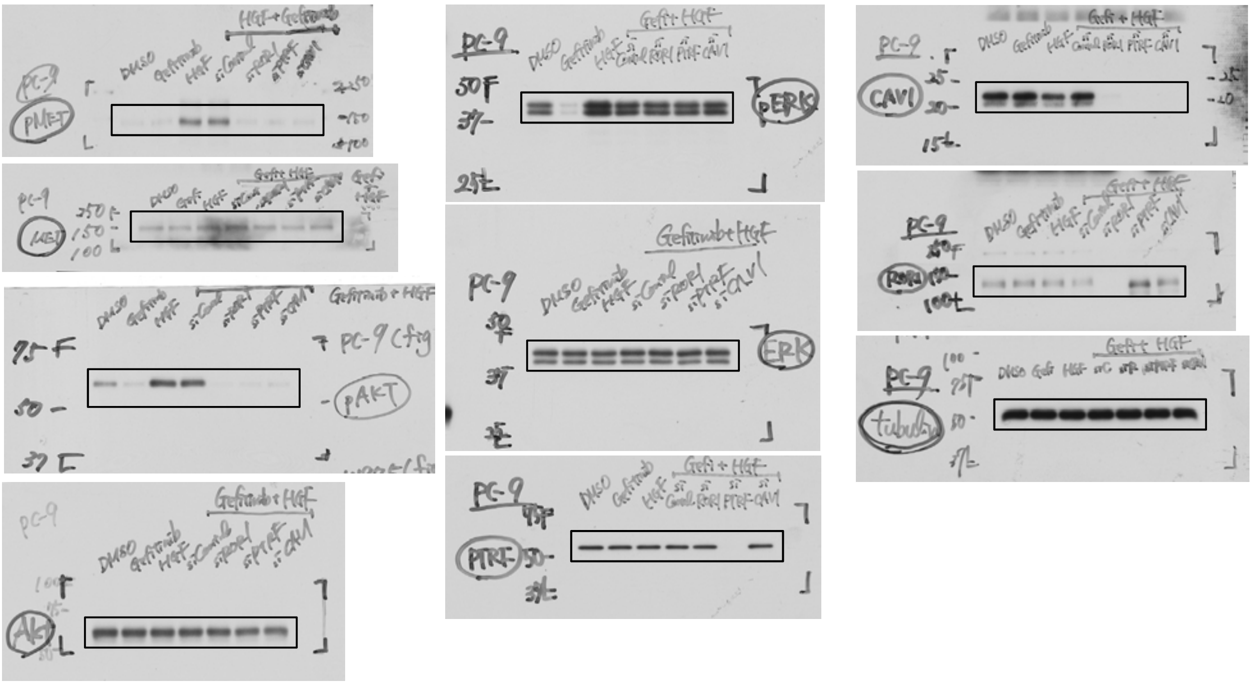

(Fig. 8c)

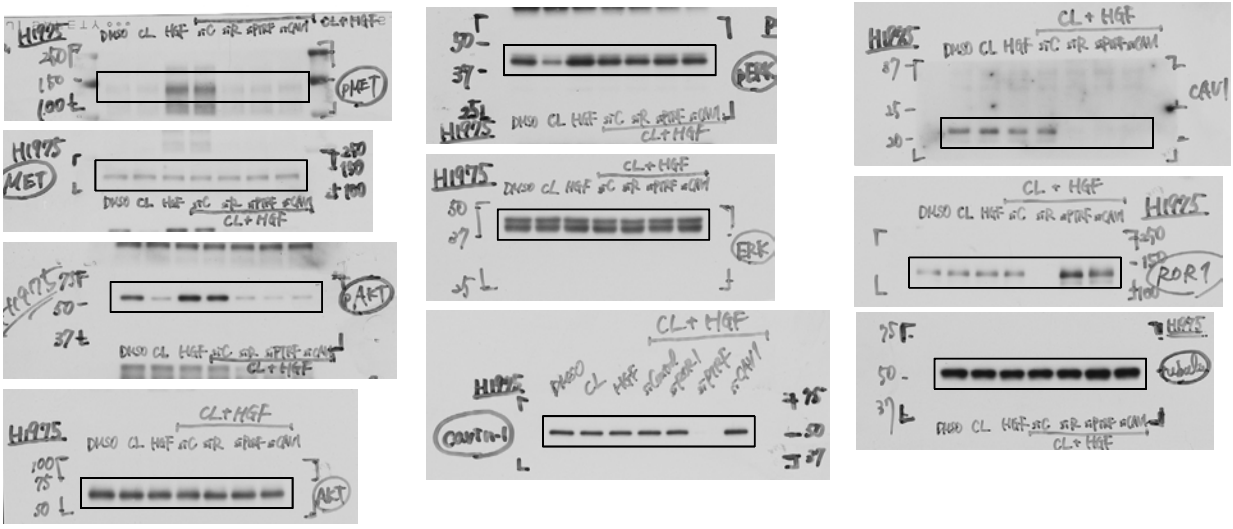

Supplementary Figure 11 | Original uncropped western blots.

Supplementary Information

(Fig. S1b) IGF-I treatment

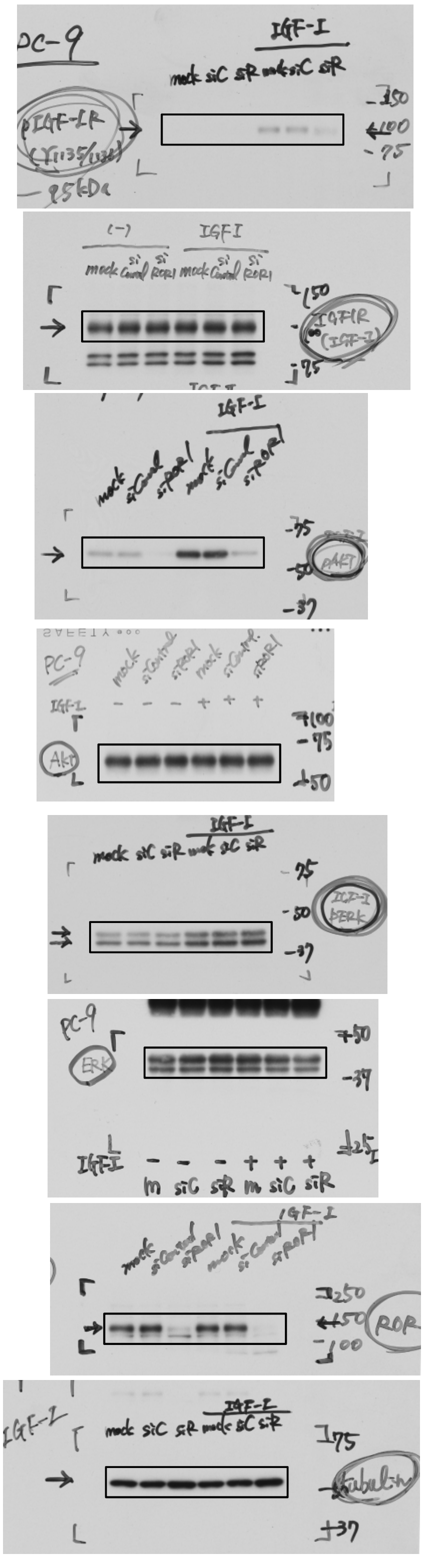

(Fig. S1b) IGF-II treatment

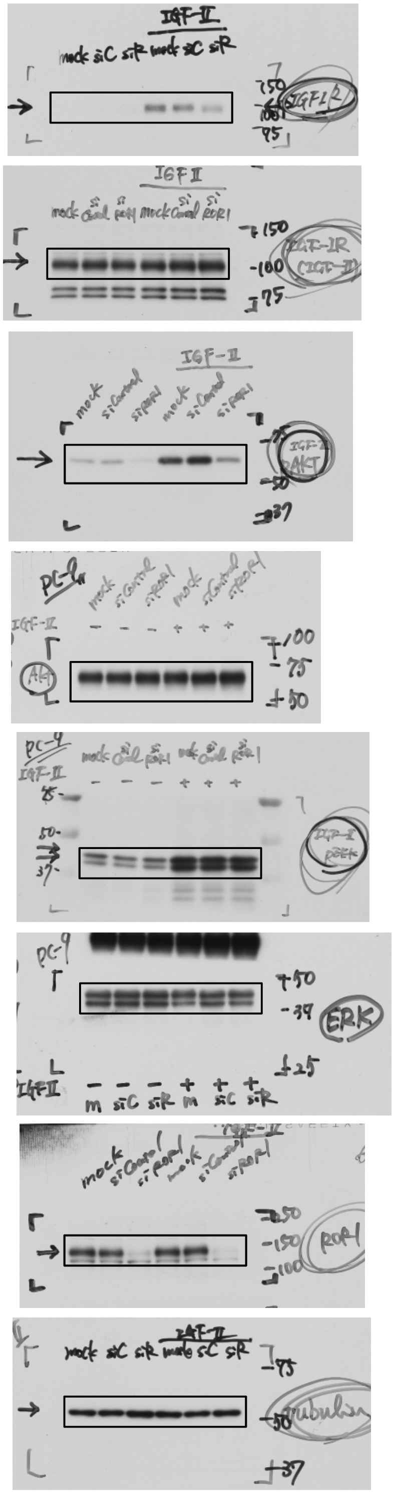

(Fig. S1b) Insulin treatment

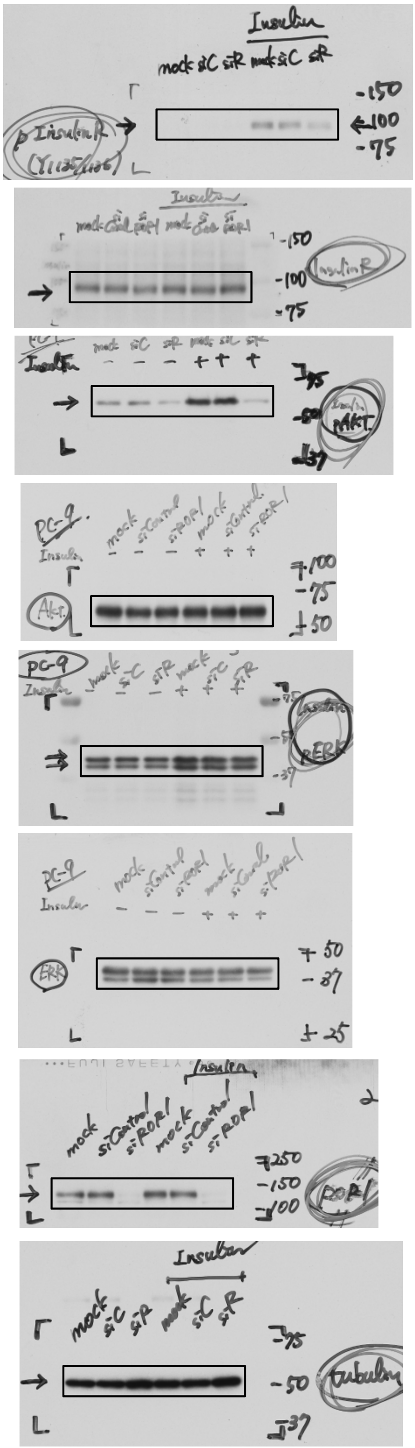

Supplementary Figure 11 | Original uncropped western blots.

Supplementary Information

(Fig. S1b) HGF treatment

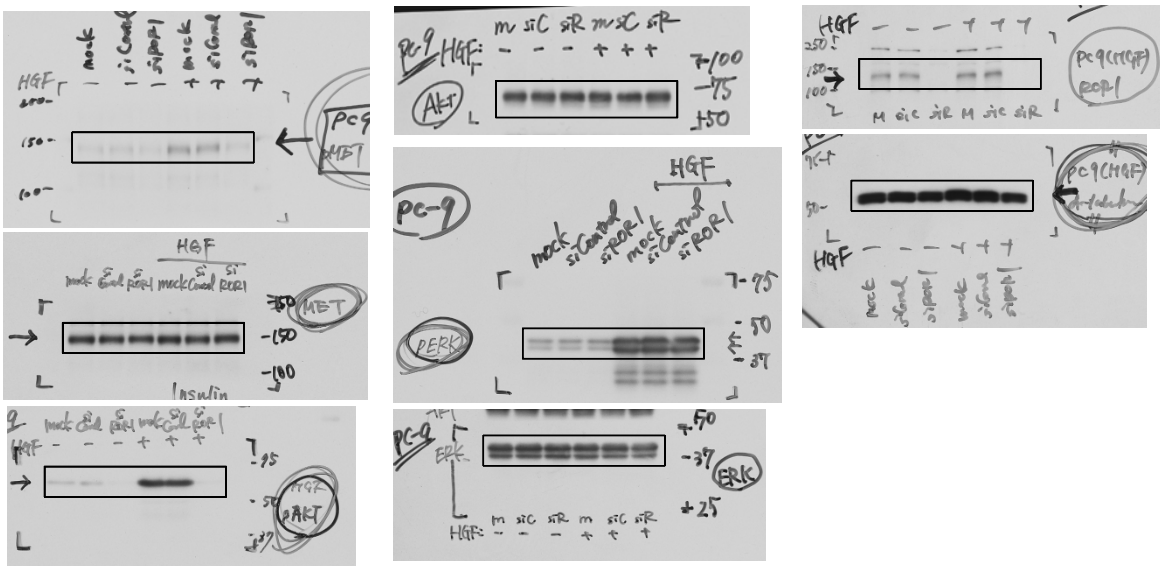

(Fig. S2d)

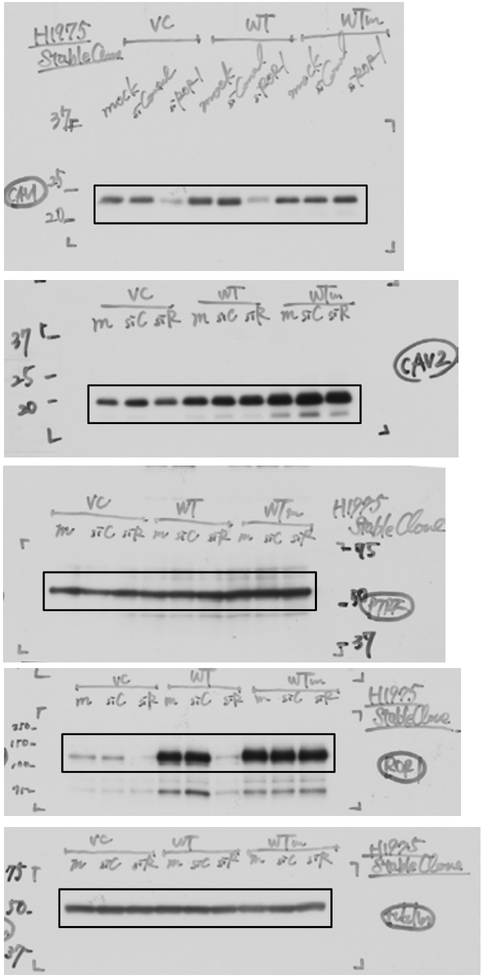

(Fig. S3a)

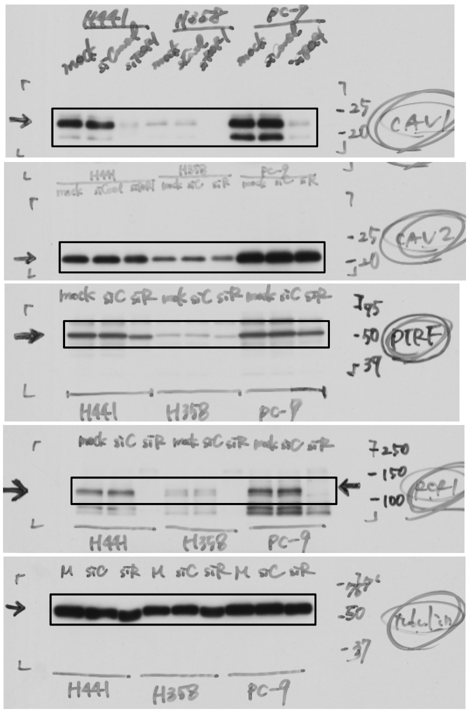

Supplementary Figure 11 | Original uncropped western blots.

Supplementary Information

(Fig. S3b) A431

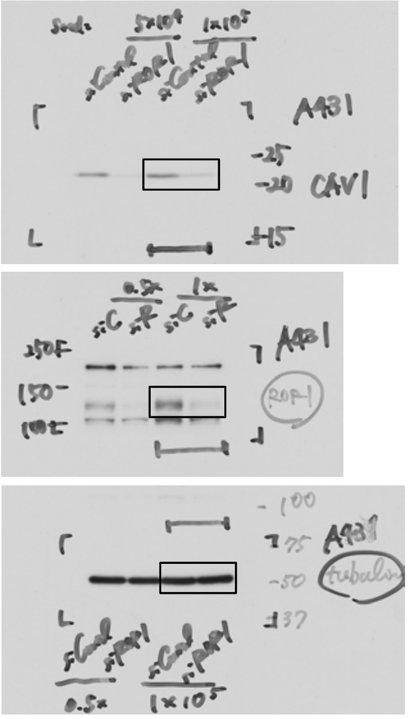

(Fig. S3b) HeLa

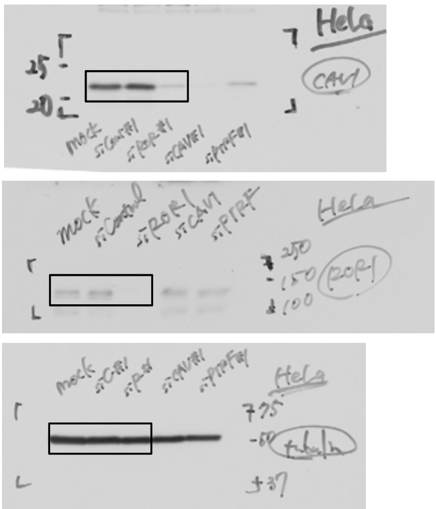

(Fig. S3c)

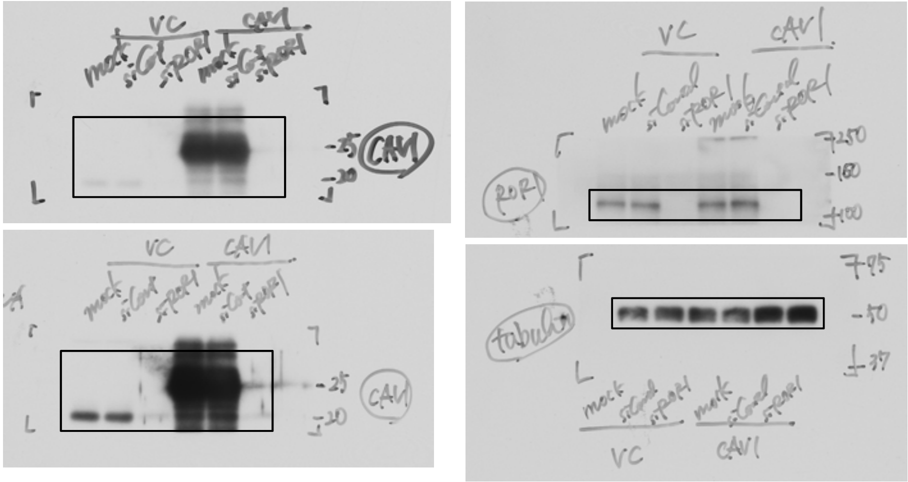

(Fig. S6a)

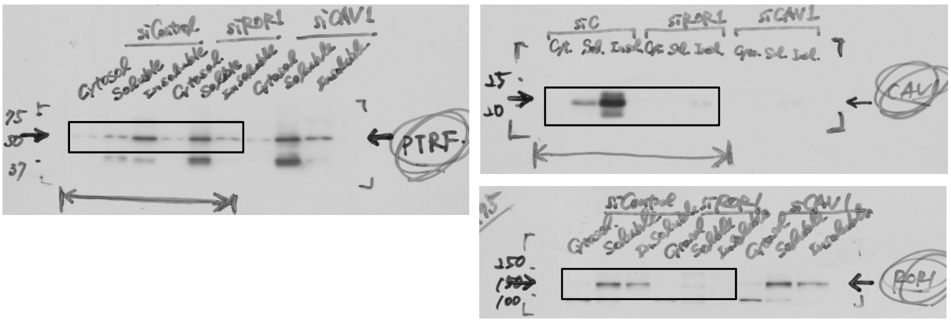

Supplementary Figure 11 | Original uncropped western blots.

Supplementary Information

(Fig. S7a)

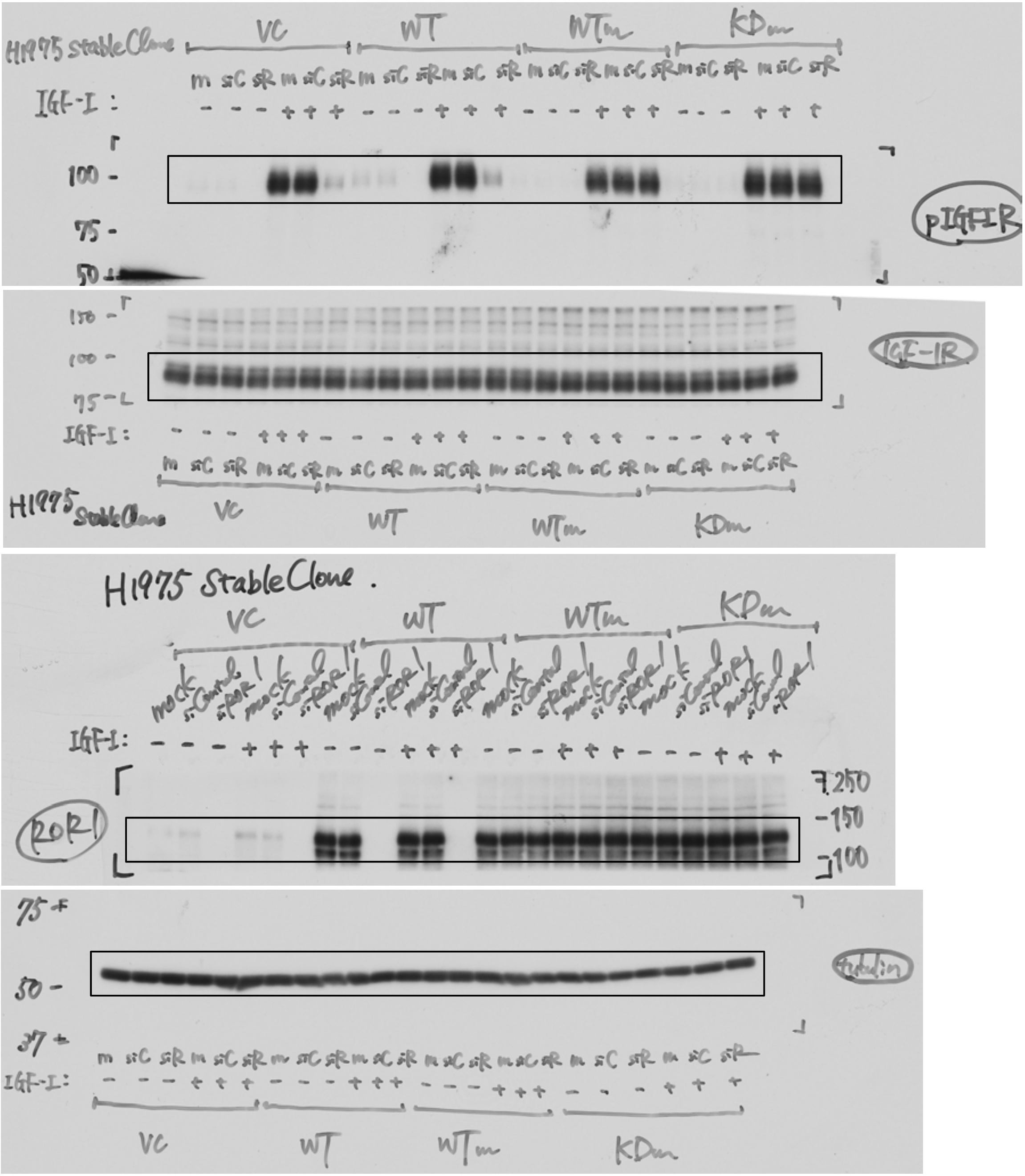

Supplementary Figure 11 | Original uncropped western blots.

Supplementary Information

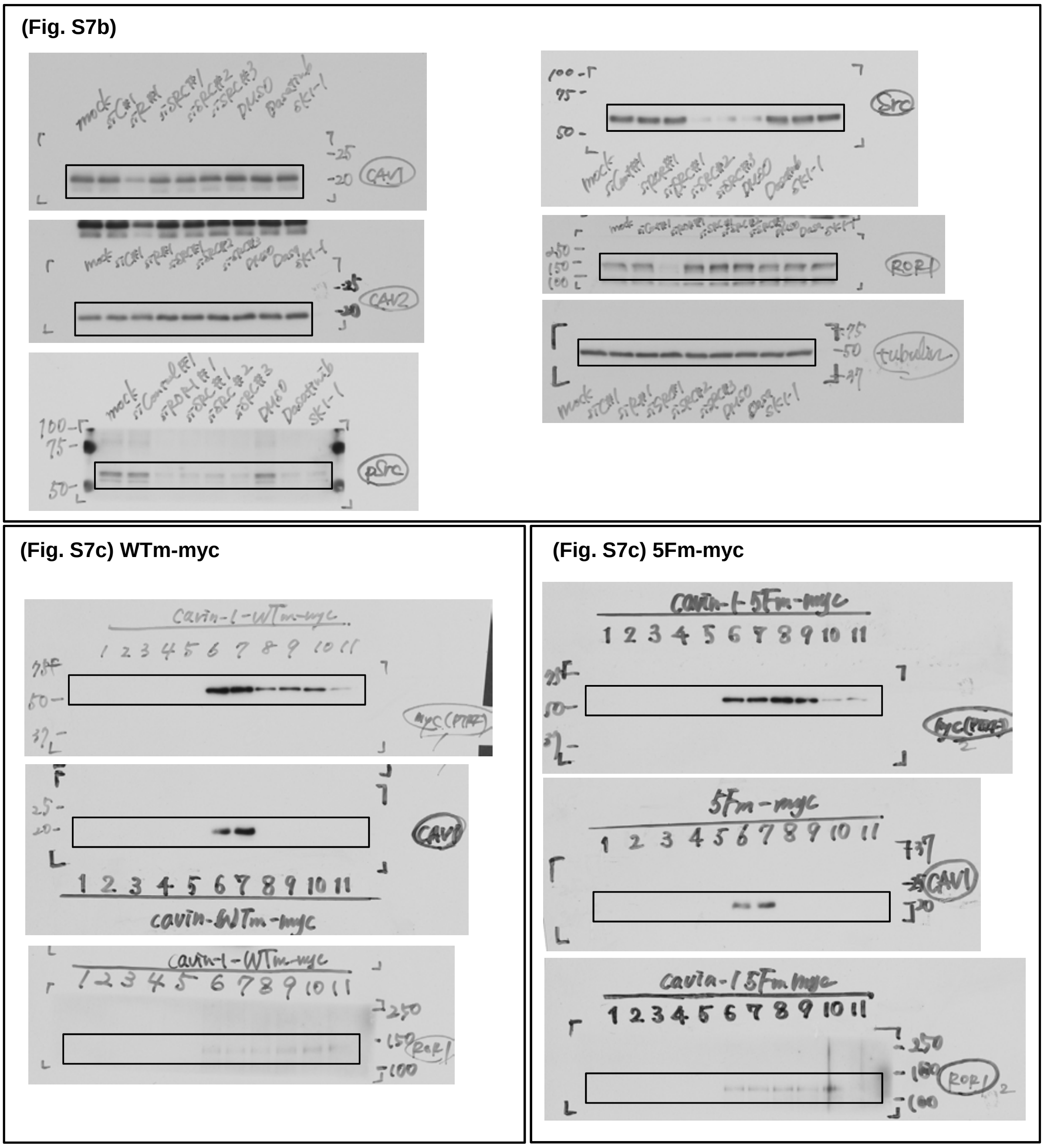

Supplementary Figure 11 | Original uncropped western blots.

Supplementary Information

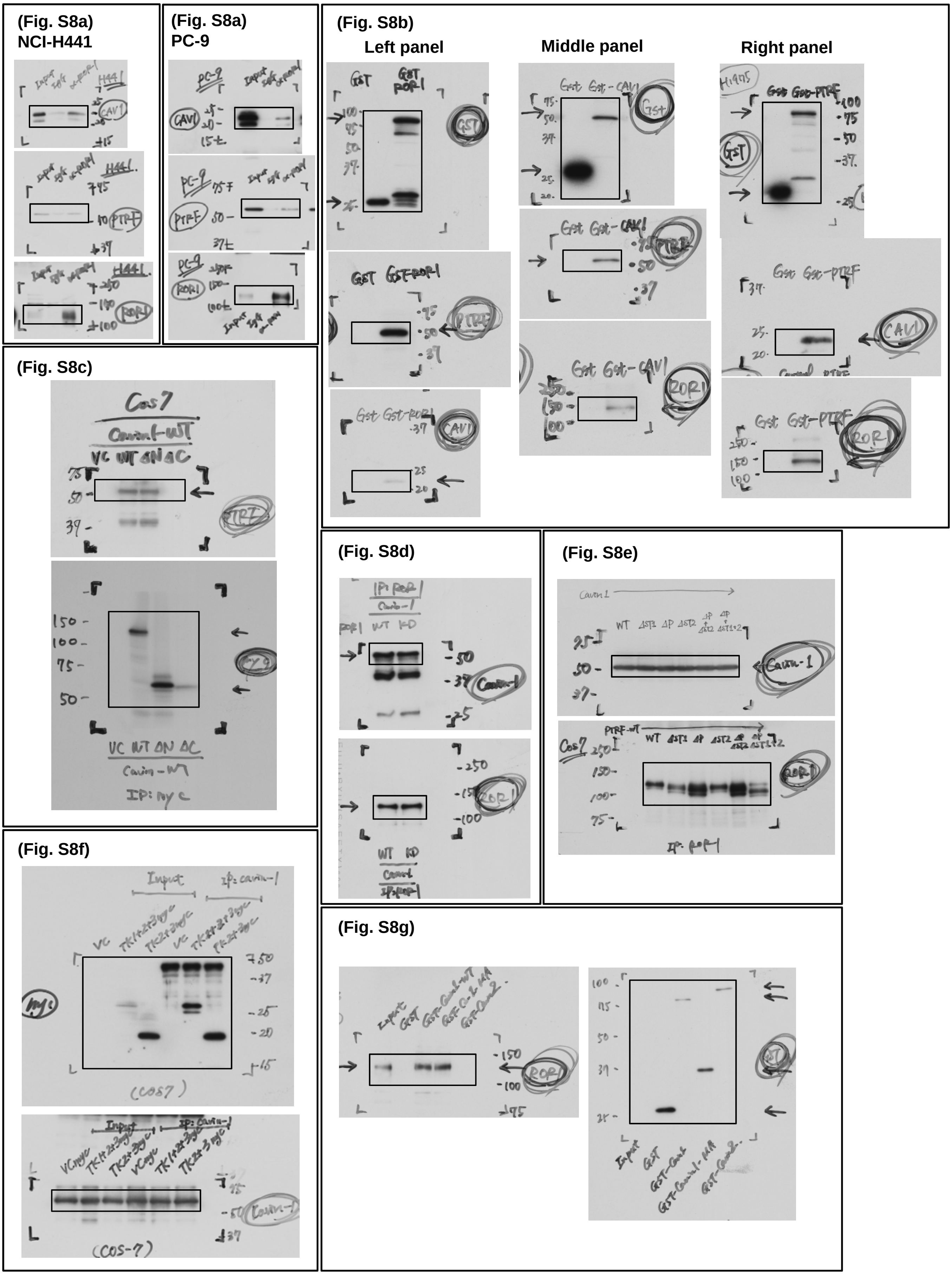

Supplementary Figure 11 | Original uncropped western blots.

Supplementary Information

(Fig. S9b) NCI-H1975

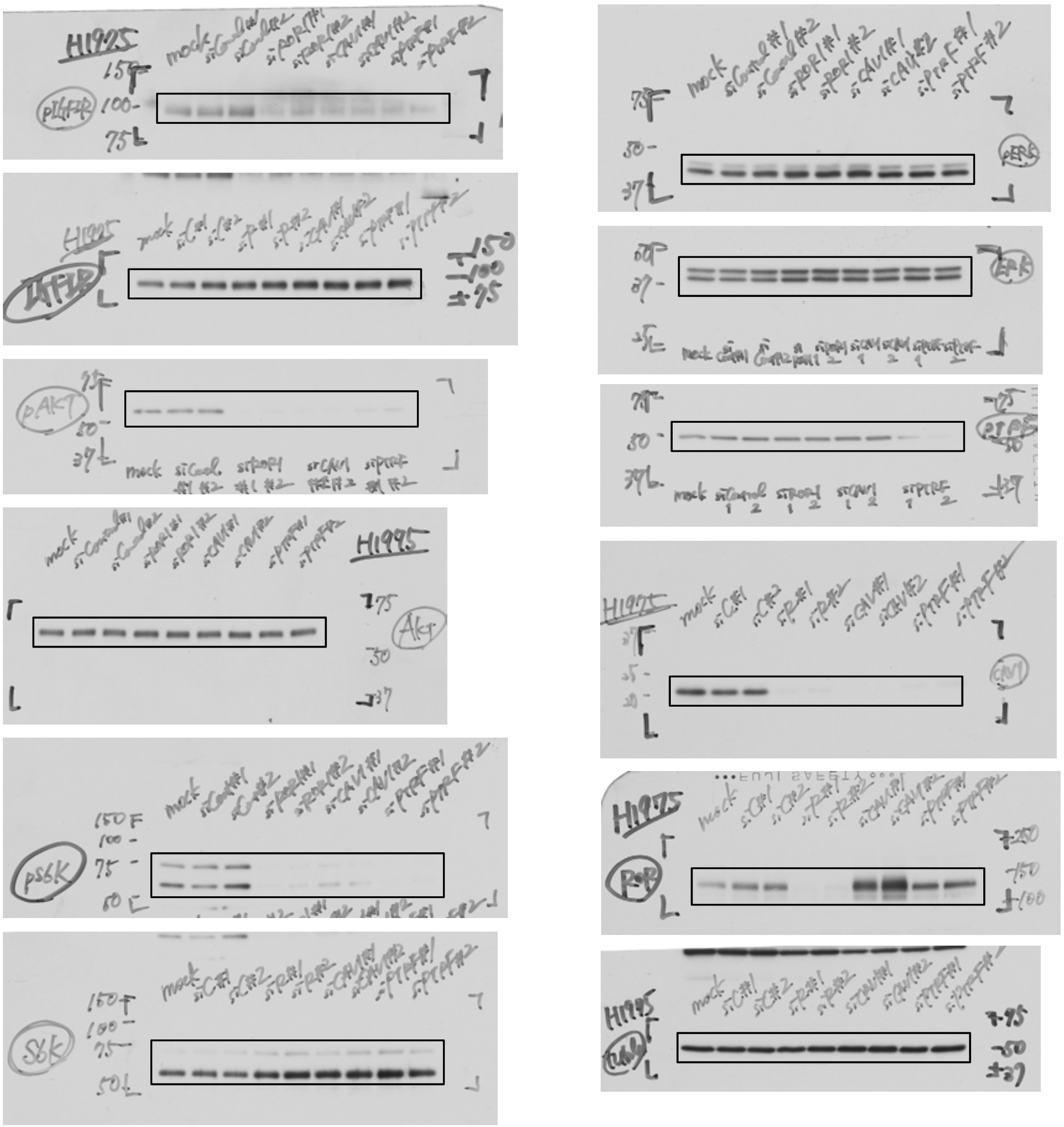

Supplementary Figure 11 | Original uncropped western blots.

(Fig. S9b) PC-9

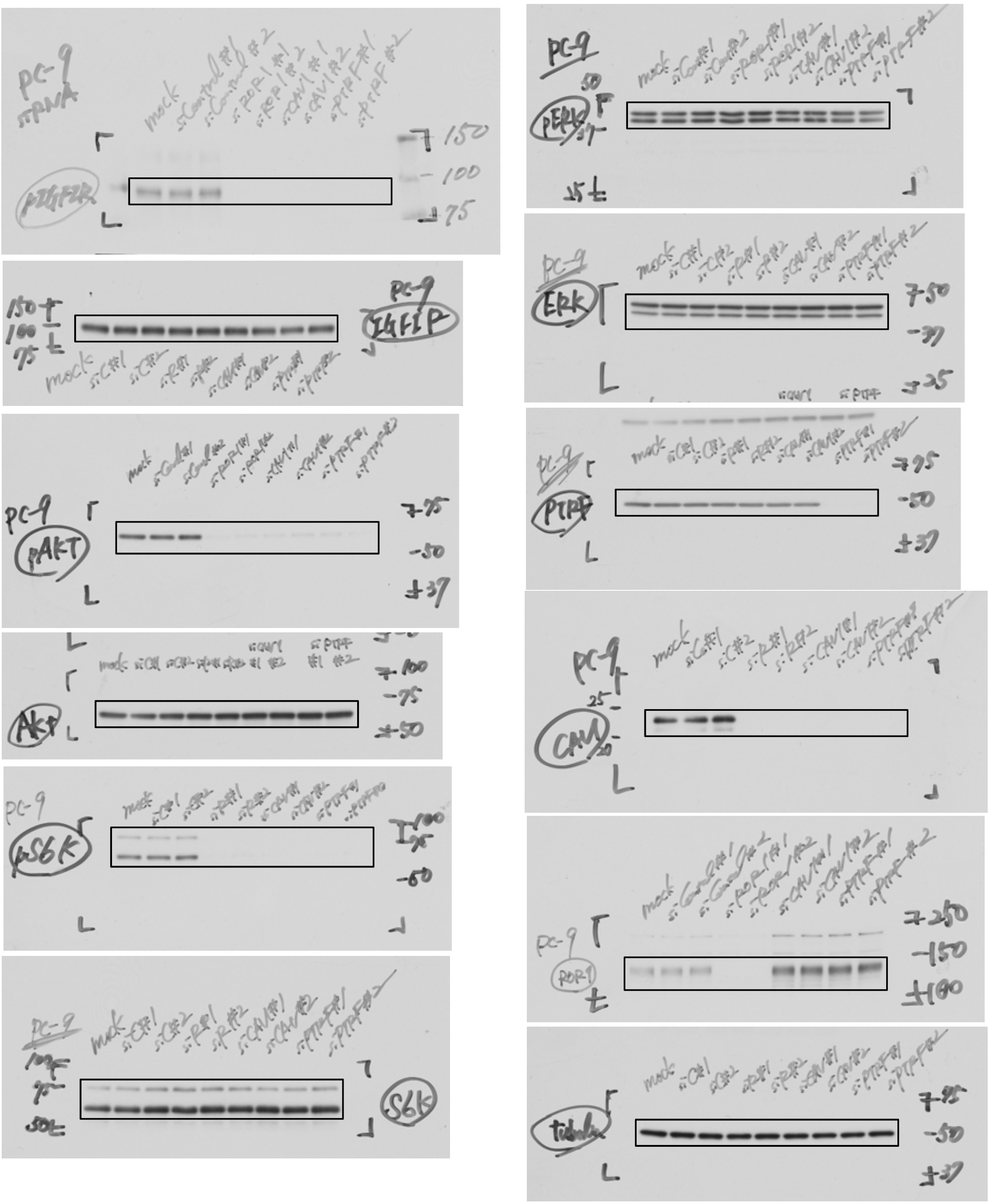

Supplementary Figure 11 | Original uncropped western blots.

Supplementary Information

Supplementary Table 1 | Primer sequences used in *in vitro* mutagenesis

ROR1 mutant primers

ROR1-KD-F	5’-GCGACCTTGAAAGACTATAACAACC-3’
ROR1-KD-R	5’-GATAGCAACCAGCTGAGCATGGTCC-3’
ROR1-TKΔ1-F	5’-CGGTCCTGGGAGGGACTCTCAAGTC-3’
ROR1-TKΔ1-R	5’-AGCAGAAAGAGGTAGCTCTTTAGCC-3’
ROR1-TKΔ2-F	5’-ATCATGAGATCCCCACACTCTGATG-3’
ROR1-TKΔ2-R	5’-GAGGAACTCATGGAGATCCCCCTGA-3’
ROR1-TKΔ3-F	5’-ATTCGCTGGATGCCCCCTGAAGCCA-3’
ROR1-TKΔ3-R	5’-GGGCAGCAAGGACTTACTCTGGACC-3’
ROR1-ΔST1-F	5’-AACCCCAGATATCCTAATTACATGT-3’
ROR1-ΔST1-R	5’-CCGAAGCCGGACGTGAATATCTTTA-3’
ROR1-ΔP-F	5’-AAGAGTCGGTCCCCAAGCAGTGCCA-3’
ROR1-ΔP-R	5’-GTTACTGAGATTACTCACTGGGCTG-3’
ROR1-ΔST2-F	5’-AATCAGGAAGCAAATATTCCTTTAC-3’
ROR1-ΔST2-R	5’-CTTGGGAGGTGGGCAGTGCTGAATC-3’

cavin-1 mutant primers

cavin-1-ΔCCD-F	5’-AGCATCAGCAAATCGCTGAAAGAGT-3’
cavin-1-ΔCCD-R	5’-CTTGATCAGCTCTTCTGAGCCGGCC-3’
cavin-1-ΔMAD-F	5’-AAATCCTTCACGCCCGACCACGTGG-3’
cavin-1-ΔMAD-R	5’-GCGGGACTCCTCAATAACCTCCTCA-3’
cavin-1-Y7F-F	5’-TTTATTGTCGAGCGGCCGCTTCCC-3’
cavin-1-Y7F-R	5’-GAGCGTGGGGTCCTCCATGGCGATC-3’
cavin-1-Y16F-F	5’-TTCCCCGACGCCGAGGCCCCGGAGC-3’
cavin-1-Y16F-R	5’-CCCGGGAAGCGGCCGCTCGAC-3’
cavin-1-Y156F-F	5’-TTCCAGGATGAAGTGAAGCTGCCGG-3’
cavin-1-Y156F-R	5’-GATCATGACTTTAAAGTTGCGGCGC-3’
cavin-1-Y308F-F	5’-TTCGCGCGCTCCAAGACCGCG-3’
cavin-1-Y308F-R	5’-CACCACGTGGTCGGGCGTGAAGGAT-3’
cavin-1-Y316F-F	5’-TTCAAGGTGCCACCCTTCACCTTCC-3’
cavin-1-Y316F-R	5’-GACCGCGGTCTTGGAGCG-3’

Supplementary Information

Supplementary Table 2 | siRNA sequences

siROR1 #1 (QIAGEN)	5'-CAGCAATGGATGGAATTTCAA-3'
siEGFR (QIAGEN)	5'-TACGAATATTAAACACTTC-3'
siERBB2 (QIAGEN)	5'-CAGAGTGATGTGTGGAGTTAT-3'
sic-MET (QIAGEN)	5'-CAACACCCATCCAGAATGTCA-3'
sicavin-1 #1 (QIAGEN)	5'-CTCCAAGACCGCGGTCTACAA-3'
sicavin-1 #2 (QIAGEN)	5'-CCCGCCGAGCGGCGCGAGAAA-3'
sic-Src #1 (QIAGEN)	5'-CTCCATGTGCGTCCATATTTA-3'
sic-Src #2 (QIAGEN)	5'-AACAAGAGCAAGCCCAAGGAT-3'
sic-Src #3 (QIAGEN)	5'-AAGCAGTGCCTGCCTATGAAA-3'
siROR1 #2 (Sigma)	5'-CCCAGTGAGTAATCTCAGT-3'
siROR1 #3 (Sigma)	5'-CCCAGAAGCTGCGAACTGT-3'
siCAV1 #1 (Sigma)	5'-AGACGAGCTGAGCGAGAAGCA-3'
siCAV1 #2 (Sigma)	5'-CCCTAAACACCTCAACGAT-3'

Supplementary Information

Supplementary Table 3 | Sequences of quantitative RT-PCR primers

ROR1	Forward primer	5'-TTCTTCATTTGCGTCTGTCG-3'
	Reverse primer	5'-GGCACACTCACCCAATTCTT-3'
CAV1	Forward primer	5'-AGCCCAACAACAAGGCCAT-3'
	Reverse primer	5'-GCAATCACATCTTCAAAGTCAATCTT-3'
CAV2	Forward primer	5'-GGCGGACGTACAGCTCTTCAT-3'
	Reverse primer	5'-GCCAGGAACACCGTCAGGAAC-3'
18S	Forward primer	5'-AATCAGGGTTCGATTCCGGA-3'
	Reverse primer	5'-CCAAGATCCAACTACGAGCT-3'
